# Integrative taxonomy reveals cryptic diversity in North American *Lasius* ants, and an overlooked introduced species

**DOI:** 10.1038/s41598-022-10047-9

**Published:** 2022-04-08

**Authors:** Sämi Schär, Gerard Talavera, Jignasha D. Rana, Xavier Espadaler, Stefan P. Cover, Steven O. Shattuck, Roger Vila

**Affiliations:** 1 Institut de Biologia Evolutiva (CSIC-UPF), Passeig Marítim de la Barceloneta, 37, 08003 Barcelona, Catalonia Spain; 2 Institut Botànic de Barcelona (IBB, CSIC-Ajuntament de Barcelona), Passeig del Migdia s/n, 08038 Barcelona, Catalonia Spain; 3 Department of Biological Sciences, The George Washington University, 800 22nd Street, NW, Suite 6000, Washington, DC 20052 USA; 4 CREAF and Unitat d’Ecologia, Facultat de Ciències, Universitat Autònoma de Barcelona, 08193 Bellaterra, Spain; 5 Museum of Comparative Zoology, Harvard University, 26 Oxford Street, Cambridge, MA 02138 USA

**Keywords:** Entomology, Invasive species, Phylogenetics, Taxonomy

## Abstract

Biological invasions are a grave threat to ecosystems. The black garden ant (*Lasius* *niger*) is a pest species in Europe. Current literature states that *L. niger* occupies a disjunct native distribution in the Holarctic, however, based on recent work, we re-evaluate this distribution. The native range of *L. niger* is reconsidered based on phylogenetic relationships (nine mitochondrial and nuclear markers, 5670 bp), DNA-barcoding (98 Holarctic specimens), morphometry (88 Holarctic specimens, 19 different measurements) and subjective assessment of phenotype. The potential spread of this species is estimated using ecological niche modeling. *Lasius niger* is more closely related to other Palearctic species than to the Nearctic ants known under this name. The latter are described as a distinct species, *L. ponderosae* sp. nov. However, DNA-barcoding discovered established populations of *L. niger* in metropolitan areas in Canada (Vancouver and Halifax). We describe a morphometrical method to delineate *L. ponderosae* sp. nov. and *L. niger*. MtDNA diversity and divergence is high within *L. ponderosae* sp. nov., but low within *L. niger.* More than 1,000,000 km^2^ are suitable as a habitat for *L. niger* in North America. This case emphasizes the critical role of integrative taxonomy to detect cryptic species and identify potential biological invasions in their nascent stages.

## Introduction

Biological invasions are a major threat to ecosystems worldwide, causing significant environmental and economic damage^1,2^. Invasive species, in particular, have been identified as a leading cause of animal extinctions^3^. Early detection and action are critical to correctly identify and halt the spread of novel invaders, but this requires up-to-date taxonomy and understanding of the geographic range extent of the species in question^4–6^. This can be challenging, particularly in groups that may include hidden (cryptic) diversity^7,8^.

Ants, in particular, can be harmful when introduced to novel ecosystems^9,10^. Five ant species are among the world’s 100 worst invasive species, representing nearly a third of the land invertebrates listed^11^. This high proportion of ants among the most problematic invasive species can perhaps be explained by their social form of organization and ecological dominance in terrestrial ecosystems^12,13^.

The North American ant fauna is well characterized at a higher taxonomic level, especially when compared to more diverse and little explored regions in the tropics^14,15^. Nevertheless, the general understanding of Nearctic ant taxonomy at the species level could still be improved for certain genera. Literature used to identify Nearctic ant species often dates back several decades (e.g., Refs.^16,17^) except for certain geographical areas (e.g., Refs.^18–20^) and taxonomic groups (e.g., Refs.^21–23^) for which more recent identification literature is available.

The ant genus *Lasius* Fabricius, 1804 is diverse and widespread across temperate and Mediterranean climatic zones of the Holarctic^24^. While the morphological diagnosis of *Lasius* species is difficult, new species are still described regularly^25–27^, and some of these are cryptic species that require complicated methods to distinguish them from one another^26^.

The common garden ant *Lasius niger* (Linnaeus, 1758) is one of the most abundant species of insects in Europe and has been rated the 7th most problematic pest animal in buildings in Britain^28^. This ant is found almost everywhere throughout Europe, except in areas with semi-arid or sub-arctic climate. Ref.^29^ discovered that *L. niger* has a widespread but overlooked sister species in the Palearctic, *L. platythorax,* ecologically replacing the former in forest habitats. *Lasius niger* is usually an inconspicuous species in undisturbed ant communities, but is often a highly dominant species in modified and anthropogenic habitats due to genomic exaptations to environmental challenges associated with urbanization^30^. This ant is most known as an agricultural pest species because of its nest building activities in farmland and greenhouses and because it supports aphids that attack crop plants^31^. Additionally, *L. niger* is considered a household and commercial pest in residential homes and businesses^32^.

Taxonomic literature holds that *L. niger* has a disjunct natural distribution, occupying both the Palearctic region and Nearctic region^17^. Within North America, however, *L. niger* has most frequently been reported from the western United States^17^. The current distribution and disjunct range of *L. niger* is puzzling and requires a taxonomic re-assessment of the ants currently described under this name in North America. Ref.^33^ hypothesized that the natural distribution of *L. niger* may not be Holarctic but is instead restricted to the Palearctic region. Here we re-evaluate the taxonomic status and distribution range of Nearctic ants identified as *L. niger* using a combination of molecular phylogenetic and morphometric data. We further study the phylogeography of the native and introduced species within the North American *L. niger-*complex. Finally, we use Environmental Niche Modelling (ENM) to predict the potential introduced range of *L. niger* in North America.

## Methods

### Phylogenetic analysis with multiple markers

We approached phylogenetic species delimitation of Holarctic ants currently identified as *"L. niger"* with a focus on North American specimens. Therefore, we compiled a phylogenetic data set representing 26 Holarctic taxa of the subgenus *Lasius*^17^ or a part of the clade of *L. niger* sensu Ref.^34^, plus two outgroup taxa (*L. pallitarsis* and *L. mixtus*). Due to the relatively high genetic divergence within ants resembling *L. niger* from the Rocky Mountains^33^, representatives of the four most divergent COI lineages were included to clarify whether they confirm monophyly of the group. The resulting DNA-alignment (5670 bp) contained sequence data originating from 9 genes (mtDNA: COI, COII, 16S, nuDNA: Defensin, H3, LR, Wg, Top1 & 28S). The data matrix containing the alignment is available from TreeBASE (http://www.treebase.org, submission ID: 29528). At least 4 genes were available for the focal taxa (*L. niger*: all 9 genes; *L. ponderosae* sp. nov.: COI, Wg, Top1 & 28S), while for outgroups completeness ranged from 1–7 genes (Supplementary Table S1). Most of these sequences were published previously^27,33,35,36^ and accessed via GenBank (Supplementary Table S1). Sequences were aligned for individual genes using MAFFT v7.453 and Geneious v.6 software. Bayesian inference was used to estimate phylogenetic relationships under the Coalescent Model in BEAST v1.8.3^37^. Partitions were set by gene and the HKY substitution model and a strict clock model with a clock rate fixed to 1.0 were chosen. BEAST was run for 10^7^ iterations in two independent runs with identical input and the log files were assessed for chain convergence and stationarity in Tracer v1.6. A second tree was calculated employing maximum likelihood (ML) in IQTREE v1.6.12^38^. Substitution models for each partition (gene) were selected by the program ModelFinder^39^ and 10,000 ultrafast bootstrap replications were performed. The following substitution models were selected: TIM2 + F + I + G4: COI & COII, TIM3 + F + G4: 16S, HKY + F + I: Defensin, HKY + F + R2: H3, Wg & 28S, K2P + I: LR & Top1.

### DNA-barcoding

DNA-barcoding data analyzed in this study represent a combination of sequence data from previous publications of the authors^27,33^ and public data from the Barcode of Life Data System (BOLD)^40^ under the name “*Lasius niger”.* A total of 72 DNA-barcodes were compiled to gather a dataset representing ants resembling *L. niger* from the Nearctic. In addition, 26 reference sequences of *L. niger* from the Palearctic were included for comparison. An overview of collection data and accession numbers for DNA-sequences for all 98 specimens is given in Supplementary Table S2. Sequences were collapsed into mitotypes using the package “haplotypes”^41^ in R v4.1.1^42^. A mitotype tree was created in IQTREE using the HKY + F + I model and 10,000 ultrafast bootstrap replications and a mitotype map was produced using the R-package “ggmap”^43^.

### Morphometry

Specimens of *L. niger* sensu Ref.^17^ from the Nearctic and Palearctic were compared morphologically. A detailed morphometric analysis was performed for 88 specimens (n = 39 Nearctic and 49 Palearctic specimens) to identify diagnostic morphometric variables between forms in both regions. For this purpose, a data set of 19 measurements originally defined by Ref.^26^ for a taxonomic revision of Palearctic *Lasius* ants was obtained for all 88 specimens. This data set contained the following variables: CL, CS, CW, dClAn, EYE, GuHL, MaDe, MP6, nGen, nGu, nHT, nOcc, nSc, nSt, PLF, PnHL, PoOc, SL and sqPDCL (See Supplementary Table S3 and Supplementary Fig. S1 for definitions of abbreviations). To find a discriminant function to distinguish Nearctic from Palearctic cryptic species, linear discriminant analysis was performed as described by Ref.^44^, after reducing the number of variables by step-wise logistic regression in R v4.1.1. In order to compare the average mesosoma profile between potential cryptic species, images were taken in lateral view and subsequently scaled to the same length and position of the thorax. The images were converted to black and white and the average image was calculated as mean darkness of pixels using the “imagematrix”-function from the ripa-package^45^ in R v4.1.1. Principal component analysis of the reduced set of 4 morphometric variables was also carried out in R v4.1.1. The native North American ants thus far known as *L. niger* were described as a new species. Type specimens are deposited at the Museum of Comparative Zoology at Harvard University (Cambridge, USA). Two paratype workers each will be deposited at the collections of University of California Davis (UCDC), the University of Utah (JTLC) and the Natural History Museum of Los Angeles County (LACM). The following standard measurements were taken from the holotype worker, 5 paratype workers and 2 gynes: Head Length (HL), Head Width (HW), Scape length (SL), Eye Length (EL), Eye Width (EW), Pronotum Width (ProW), Mesosoma Length (ML), Hind Tibia Length (HTL), Cephalic Index (CI) and Scape Index (SI).

### Ecological niche modelling

In order to estimate the suitable habitat of the introduced *L. niger* in North America, we carried out ecological niche modeling using the R package biomod2^46^, based on 180 presences and 182 absences from the Old World. We subsequently projected the resulting model to North America. Presence points were obtained from reviewed literature and websites (Supplementary Table S4). Absence points were extracted from areas outside the climatic envelope of *L. niger*: this species is absent from the dry regions of Iberia, the Mediterranean coast, the Balearic Islands, Asia Minor^47^, the islands of Crete, Sardinia and Sicily^48^, Africa, the Middle East, the Arabian Peninsula and boreal, subarctic and arctic parts of Scandinavia and Russia^49^. In addition, a number of absences were selected for sites where authors have noted local or regional absence of *L. niger* during myrmecological surveys. All 19 WorldClim climatic layers^50^ and the freely available Global Land Cover Map for 2009^51^ were used as explanatory variables. The following models were explored: GLM, GAM, MARS, CTA, FDA, GBM and RF, and a total of 20 runs were performed. Models were evaluated using the TSS, ROC, and KAPPA-method. The results from all models were combined using ensemble modeling. Models were finally projected to North America using the function “BIOMOD_EnsembleForecasting” in biomod2. Areas of suitable habitat in Europe and North America were divided into three ranges of occurrence probability: 0.2–1, 0.5–1 and 0.8–1, on a probability scale of 0–1. Area sizes for each occurrence probability range were approximated by multiplying the number of raster cells with values above the defined probability threshold with the median cell size in km^2^.

## Results

### Phylogenetic analysis with multiple markers

**Figure 1:**
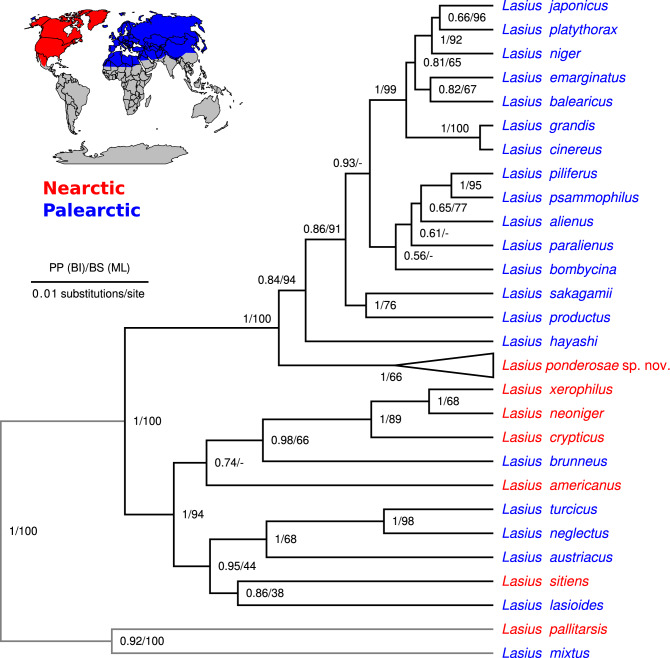
Molecular phylogeny of 26 Holarctic ant taxa belonging to the subgenus *Lasius* sensu Wilson (1955) and two outgroup taxa (*L. pallitarsis* and *L. mixtus*). The phylogeny was calculated under the coalescent model and incorporates data from 9 genes (mtDNA: COI, COII, 16S, nuDNA: Defensin, H3, LR, Wg, Top1 & 28S). Names of species native to the Nearctic are shown in red and those of species native to the Palearctic in blue. Node labels show posterior probability (Bayesian inference) followed by bootstrap support (Maximum likelihood). The scale bar indicates the length of 0.01 substitutions/site.

The final alignment of 5670 bp length contained 843 variable sites (14.7%). Missing data accounted for 53.5% of the alignment cells and the relative GC content was 39.5%. Our phylogeny suggests that the investigated Holarctic taxa of the *niger* clade sensu Ref.^34^ are divided into two major clades with strong statistical support (Fig. 1). The first major clade (*L. niger* group) consists exclusively of Palearctic species (*L. niger, L. platythorax, L. japonicus, L. emarginatus, L. balearicus, L. grandis, L. cinereus,* the *L. alienus*-complex, *L. sakagamii, L. productus* and *L. hayashi*), with the exception of an unnamed Nearctic subclade recovered as sister to the rest of the group. This unnamed subclade we describe as a new species below (*L. ponderosae* sp. nov.). *Lasius ponderosae* sp. nov. corresponds to what was previously known as the Nearctic form of “*L. niger*” sensu ref.^17^, but includes some western Nearctic populations formerly assigned to “*L. alienus”*^17,52^ as well. Monophyly of *L. ponderosae* sp. nov. was fully supported by Bayesian inference (pp = 1) and moderately supported by maximum likelihood (66% bootstrap support, Fig. 1). *Lasius ponderosae* sp. nov. is distantly related to *L. niger*; and *L.niger* is a close relative of *L. japonicus* and *L. platythorax,* as well as other Palearctic taxa. The second major clade (*L. brunneus* group) within the investigated Holarctic members of the *L. niger* clade contains both Nearctic and Palearctic species not closely related to the taxa of interest (Fig. 1).

### DNA-barcoding

**Figure 2:**
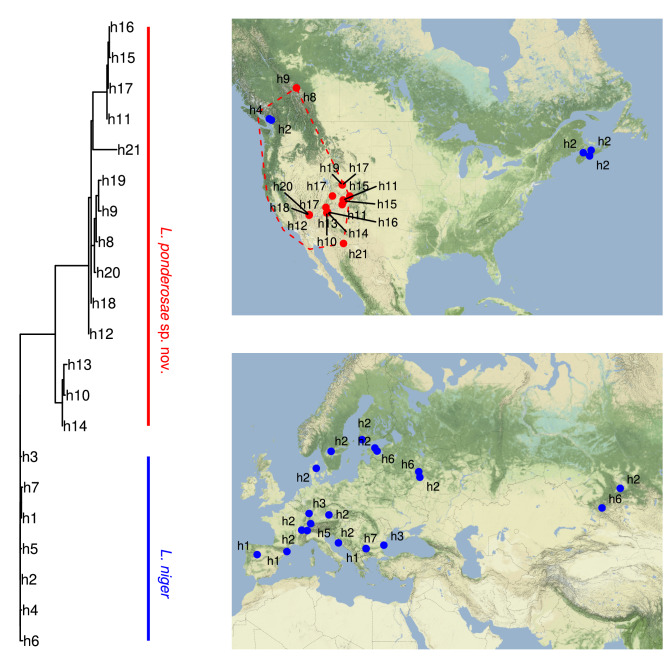
Mitotype tree and distribution maps for 98 DNA-barcodes belonging to 7 mitotypes of the ant *Lasius niger* (blue, n = 70) and 15 mitotypes of *L. ponderosae* sp. nov. (red, n = 28). The red dashed line delimits the expected natural range of *L. ponderosae* sp. nov.^53^ Maps have been created using the free R-package “ggmap” v3.0.0 (https://github.com/dkahle/ggmap) in R v4.1.1. Map tiles by Stamen Design, under CC BY 3.0.

The native North American species *L. ponderosae* sp. nov. contains at least 15 COI-mitotypes (n = 28 sequenced specimens) belonging to four distinct deep lineages, with divergences of up to 5.9%. Haplotype diversity was 0.899 and nucleotide diversity was 0.012. None of the mitotypes of this species was found to be widespread or particularly abundant. In striking contrast, low genetic diversity was found in *L. niger* across its entire distribution (Fig. 2). No more than 7 different COI-mitotypes were detected in samples from distant localities representing most of the known range (n = 70 specimens from 12 countries), from Spain in the West to the Siberian Baikal-region in the East (Fig. 2). Their maximum pairwise divergence was only 0.6%, with a haplotype diversity of 0.682 and a nucleotide diversity below 0.001. One mitotype of *L. niger* is highly dominant within the native range, occurring from Western Europe to Central Siberia (mitotype h2 in Fig. 2).

### Recent Palearctic *L. niger* introduction to Canada

Palearctic *Lasius niger* was introduced to several localities in coastal Canada in recent times, where at least 11 populations were found in two metropolitan areas (Vancouver and Halifax areas, see Table S2 for details). Those populations consist of the most dominant Palearctic mitotype of *L. niger* (h2). However, in 3 localities in the Vancouver area, 3 specimens with a second mitotype were found (mitotype h4, Fig. 2, Table S2) in syntopy with those carrying the most common mitotype h2. This second Canadian COI-mitotype (h4) was not found among our samples from the Old World, although it only differs by a single nucleotide substitution from mitotypes found there. A review of BOLD data revealed that the Canadian barcoded specimens of *L. niger* were mostly collected in anthropogenic habitats such as schoolyards (Supplementary Table S2).

### Description of *Lasius ponderosae* sp. nov

*Lasius ponderosae* Schär, Talavera, Rana, Espadaler, Cover, Shattuck and Vila. ZooBank LSID: urn:lsid:zoobank.org:act:22E2743A-2F1C-4870-B318-A1F2DF2B464C 

*Etymology*: *ponderosae* alludes to the ponderosa pine tree (*Pinus ponderosa*) that is at the centre of occurrence in the ponderosa pine—gambel oak communities in the western Rocky Mountains and northern Arizona.

*Type material*: located at the Museum of Comparative Zoology, Cambridge, USA. Two paratype workers each will be deposited at the collections of University of California Davis (UCDC), the University of Utah (JTLC) and the Natural History Museum of Los Angeles County (LACM).

**Figure 3:**
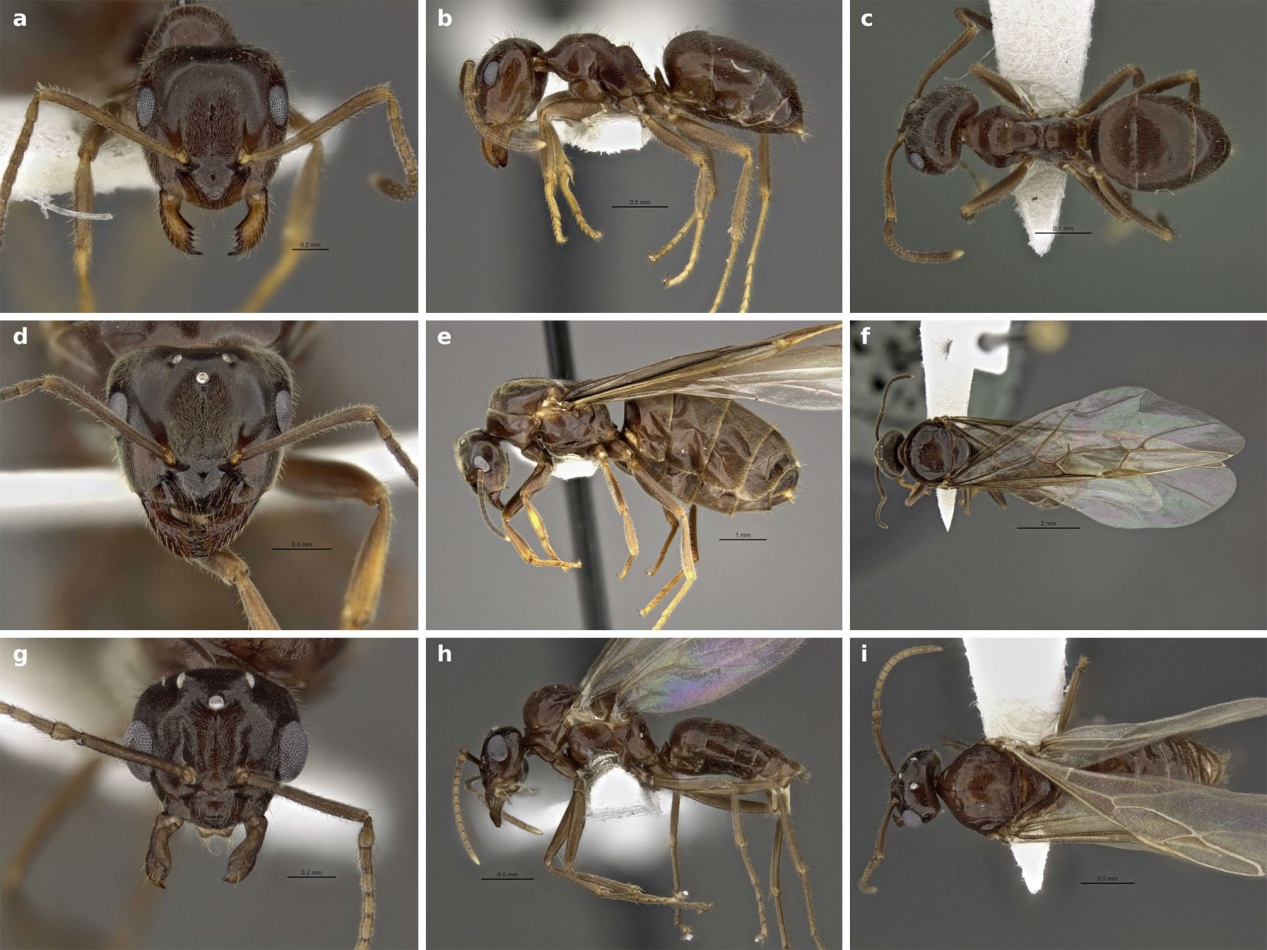
Frontal, lateral and dorsal view of the holotype worker (**a**–**c**), a paratype gyne (**d**–**f**) and a paratype male of *Lasius ponderosae* sp. nov. (**g**–**i**).

*Holotype*: worker, Fig. 3a–c. Type locality: USA, Utah: Uintah Co., Uintah Mtns., 2408 m. 18.6 mi N. Jct. Rt. 40 on Rt. 191, 40.66378°N, − 109.47918°E, leg. 15.VII.2013, S. P. Cover; J. D. Rana, collection code SPC 8571. Measurements [mm]: HL: 0.899, HW: 0.823, SL: 0.821, EL: 0.239, EW: 0.189, ProW: 0.56, ML: 1.069, HTL: 0.863, CI: 92, SI: 100.

*Paratypes*: 15 workers, two gynes (Fig. 3d–f), two males (Fig. 3g–i) from the same series as the holotype, morphometric data is given in the Appendix, Table S5 and Table S6. CO1 mitotype h17: Genbank Accession no. LT977508.

**Figure 4:**
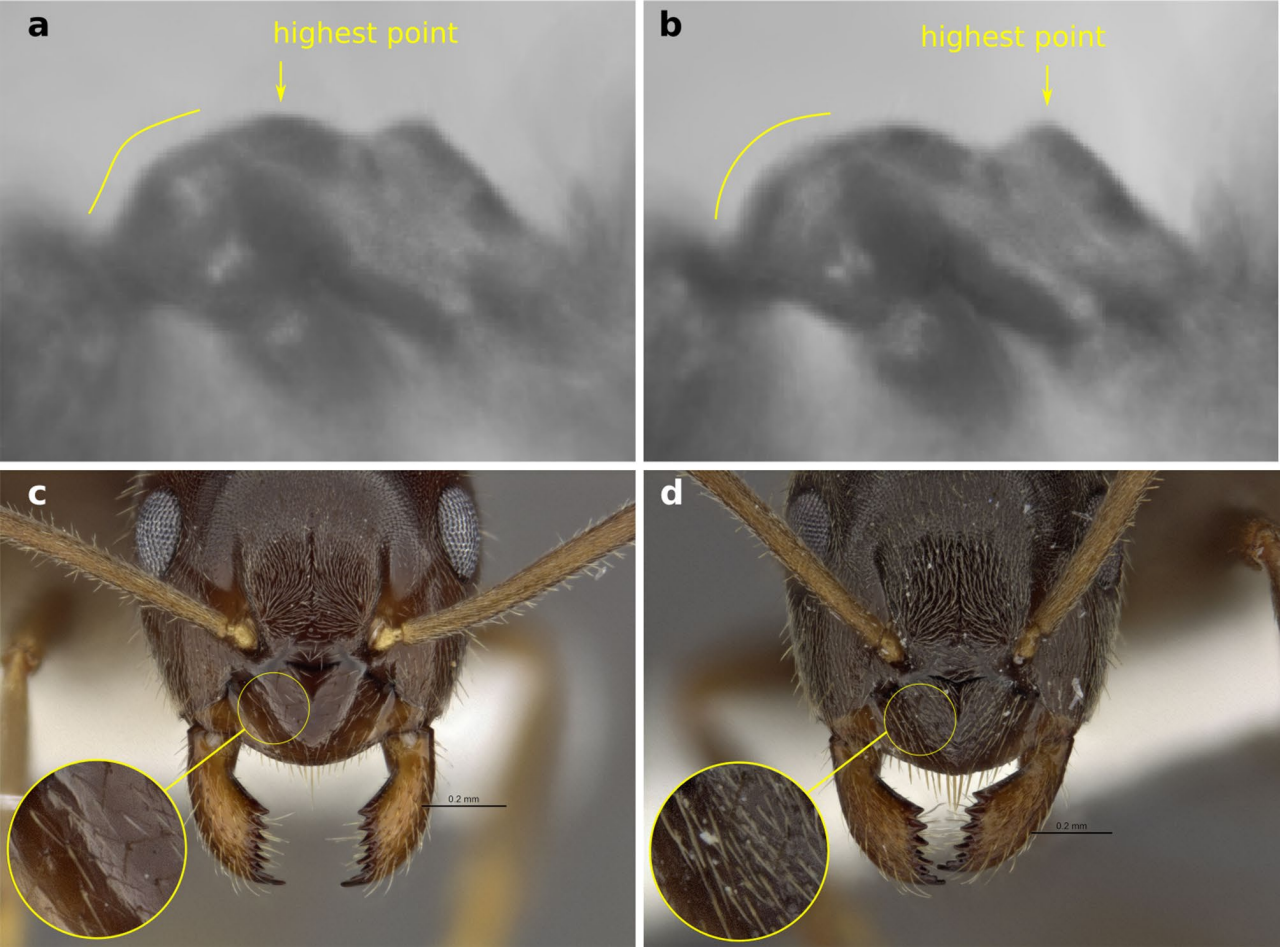
Average thorax profile of *Lasius ponderosae* sp. nov. (**a**) and members of the Palearctic *L. niger-*complex (**b**). Figures were created by image averaging (*L. ponderosae* sp. nov n = 35; Palearctic *L. niger-*complex n = 30 specimens). Frontal view of head and detail of clypeus of the Holotype worker of *L. ponderosae* sp. nov. (**c**) and a non-type worker of *L. niger* (**d**).

*Description of the worker caste:* A member of a complex of cryptic species resembling *L. niger*. Intermediate in overall body size, antennal scape length and eye size and comparable to related species (Table 1). Terminal segment of maxillary palps and torulo-clypeal distance relative to head size shorter than in related Palearctic species (Table 1). Mandibles with 8 or rarely 7 or 9 regular denticles and lacking offset teeth at their basal angle. Penultimate and terminal basal mandibular teeth of subequal size, and the gap in between with subequal area than the basal tooth. Anterior margin of clypeus evenly rounded. Dorsofrontal profile of pronotum slightly angular (Fig. 4a). Propodeal dome short and flat, usually lower than mesonotum (Fig. 4a). Body with abundant and long pilosity, especially lateral propodeum, genae, hind margin and underside of head. Pilosity of tibiae and antennal scapes variable, ranging from almost no setae ("*L. alienus*"-like phenotype) to very hairy ("*L. niger*"-like phenotype). Microscopic pubescent hairs on forehead between frontal carinae long and fine. Clypeus typically with only few scattered pubescent hairs (Figs. 3, 4c). Coloration of body dark brown, occasionally yellowish- or reddish-brown or slightly bicolored with head and thorax lighter than abdomen. Femora and antennal scapes brown. Mandibles and distal parts of legs yellowish to dark brown. Specimens of all 3 castes are shown in Fig. 3a–i and morphometric data are summarized in Table 1 and raw measurements are available in Table S5 and S6.

**Table 1:** Morphometric data of *Lasius ponderosae* sp. nov. and comparison to morphologically similar Palearctic species.

Variable	*Lasius ponderosae* sp. nov., N = 39^1^	Palearctic *L. niger-*complex, N = 49^1^	p-value^2^
CW [mm]	0.81 [0.66, 0.90]	0.92 [0.66, 1.09]	< 0.001
CL [mm]	0.88 [0.73, 0.97]	0.98 [0.75, 1.15]	< 0.001
CS [mm]	0.84 [0.70, 0.94]	0.95 [0.71, 1.12]	< 0.001
CL/CW	1.077 [1.026, 1.143]	1.062 [0.997, 1.162]	0.043
SL/CS	0.97 [0.92, 1.03]	0.98 [0.90, 1.06]	0.2
sqPDCL [μm]	5.47 [3.90, 7.37]	4.25 [3.58, 6.11]	< 0.001
PoOc/CL	0.242 [0.212, 0.262]	0.244 [0.209, 0.271]	0.058
nGen	13.0 [5.0, 19.0]	8.5 [3.0, 18.0]	< 0.001
EYE/CS	0.239 [0.220, 0.259]	0.237 [0.213, 0.266]	0.5
nGU	13.0 [4.0, 24.0]	8.0 [2.0, 25.0]	< 0.001
nOCC	18.5 [9.0, 26.0]	16.0 [7.0, 25.0]	0.002
GUHL/CS	0.117 [0.071, 0.150]	0.084 [0.055, 0.128]	< 0.001
MaDe			0.001
7	6 (19%)	0 (0%)	
8	26 (81%)	32 (86%)	
9	0 (0%)	5 (14%)	
Unknown	7	12	
dCLAN/CS	0.044 [0.026, 0.056]	0.051 [0.042, 0.061]	< 0.001
MP6/CS	0.161 [0.133, 0.206]	0.174 [0.136, 0.229]	< 0.001
PnHL/CS	0.134 [0.090, 0.190]	0.127 [0.067, 0.171]	0.091
nSt	7.00 [4.00, 11.00]	4.00 [0.00, 10.00]	< 0.001
nHT	26 [11, 35]	18 [6, 39]	< 0.001
nSC	25 [4, 39]	18 [10, 36]	< 0.001
PLF [mm]	0.034 [0.026, 0.048]	0.028 [0.021, 0.041]	< 0.001

For a definition of variables see Table S3, Fig. S1. These measurements were originally defined by Ref.^26^.

^1^Median [Range]; n (%).

^2^Wilcoxon rank sum test; Fisher's exact test.

*Diagnosis: Lasius ponderosae* sp. nov. workers key out to "*L. niger*" using Wilson's 1955 key to the Nearctic *Lasius* species. However, some populations with reduced pilosity may also be identified as "*L. alienus*" using this key. *Lasius alienus* is a Eurasian species not known from North America^33^. The Nearctic "*L. alienus*" sensu Wilson (1955) includes both, *L. americanus* as well as populations of *L. ponderosae* sp. nov. with sparse setae counts on tibia and/or scapes. *Lasius ponderosae* sp. nov. can be distinguished from *L. americanus* by the presence of abundant, long setae surpassing the sides of the head in full face view (nGen > 5 and nOcc > 10 vs. nGen < 5 and nOcc < 10 in *L. americanus*). Distinguishing *Lasius ponderosae* sp. nov. from related Eurasian species (e.g., *L. niger* or *L. platythorax*) by subjective eye inspection is difficult because there are no easily visible morphological traits allowing a separation of *Lasius ponderosae* sp. nov. from all these taxa. *Lasius ponderosae* sp. nov. is therefore a cryptic species. For *L. niger,* introduced to North America, nest samples can often be distinguished from *L. ponderosae* sp. nov. using the average mesosoma profile (Fig. 4a,b) and by fewer pubescent hairs on the clypeus (Fig. 4c,d). A distinction between single workers of *L. ponderosae* sp. nov. and *L. niger, L. platythorax* and related Palearctic species can be achieved by calculating the following discriminant function with measurements taken in mm:

*D* =  − 43.792*GUHL + 113.436*dCLAN + 75.68*MP6  − 0.431*nSt  − 10.075.

**Figure 5:**
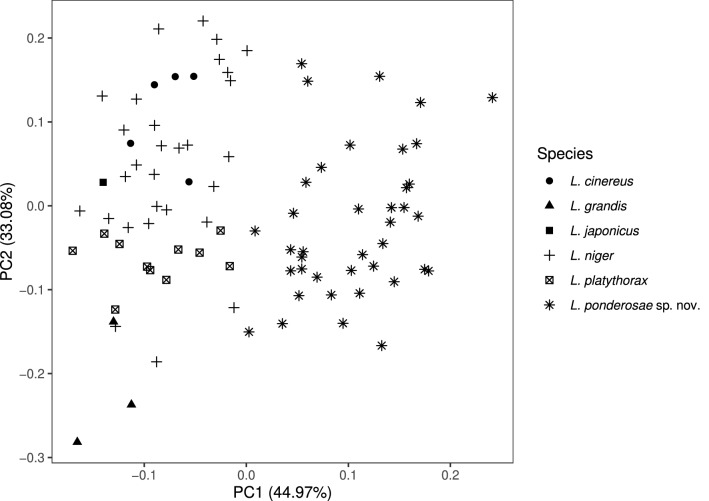
Principal component plot for the 4 most diagnostic morphometric variables (GUHL, dCLAN, MP6 and nSt) to distinguish individual specimens of *Lasius ponderosae* sp. nov. (n = 39) from those belonging to morphologically similar-looking Palearctic species (n = 49). For a definition of variables see Supplementary Table S3 and Fig. S1.

Negative values of *D* indicate *L. ponderosae* sp. nov. (*L. ponderosae* sp. nov., n = 39, median, range: − 3.18 [− 5.83, − 0.43]; *L. niger, L. platythorax, L. grandis, L. japonicus* and *L. cinereus*, n = 49, median, range: 2.67 [0.18, 4.61]). A principal component analysis plot for the four most diagnostic variables (GUHL, dCLAN, MP6 and nSt) is shown in Fig. 5. For a definition of the variables used in the function, see Supplementary Table S4 and Supplementary Fig. S1. All morphometric data are available in Table S6.

*Distribution:* Western North America: Alberta, Arizona, Baja California, California, Colorado, Idaho, Montana, Nevada, Oregon, Utah and Washington^53^.

*Habitat*: Occurring in a broad range of habitats and elevations, from 20–3220 m a. s. l. (mean: 1997 m)^53^. Typical for mid-elevations in the mountains, whose primary habitat is ponderosa pine forest and its associated communities (e.g., dry meadows, sagebrush, gambel oak woodland) or at higher elevations in meadows bordered by aspen-spruce or alpine scree slopes, but also in other habitats. Nests in and under dead wood, and under stones in soil^53^.

### *Lasius niger* niche modeling and potential spread in North America

**Figure 6:**
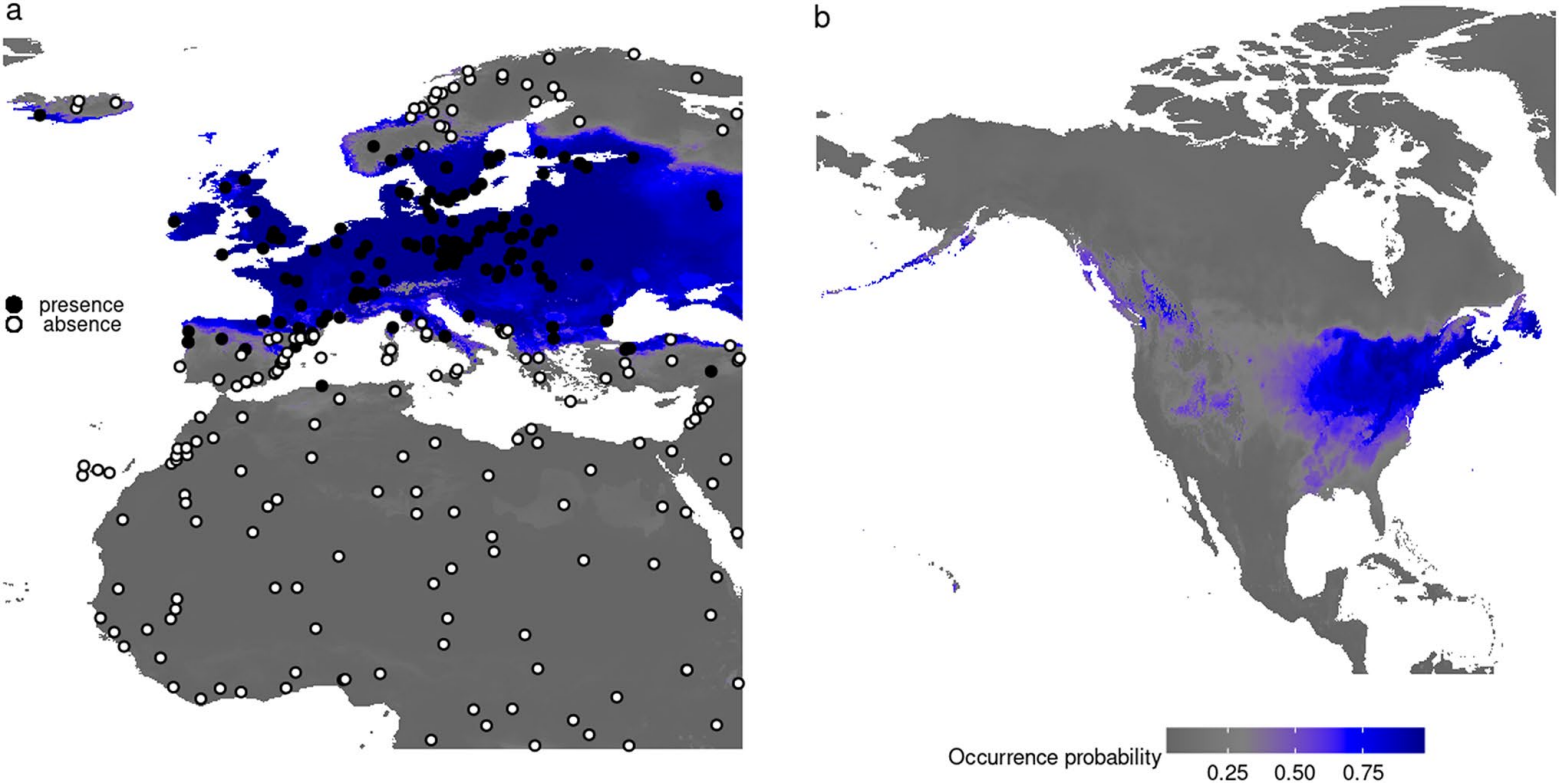
Projected occurrence probability from ecological niche modeling for the Palearctic ant *Lasius niger* which has been introduced to Canada, based on 19 climatic and one land use variable. The intensity of blue colour indicates the probability of occurrence on a 0–1 scale based on 180 presences (black circles) and 182 absences (white circles) in the native range in the Old World (**a**). The model was then projected to North America to estimate areas of suitable habitat for this introduced species (**b**). These maps have been created using the free R-package “ggplot2” v3.3.5 (https://ggplot2.tidyverse.org) in R v4.1.1.

According to the ROC, TSS and kappa statistics, the tested modeling techniques displayed good performances for *L. niger* (mean values of ROC > 0.9, TSS and kappa > 0.8 across models and runs). The strongest predictors were: Annual Mean Temperature (mean variable importance = 0.32), Mean Temperature of Coldest Quarter (0.23), Temperature Annual Range (0.23) and Temperature Seasonality (0.24). The contribution of land cover was low (0.02). The model predicted high probabilities of occurrence of *L. niger* in the eastern United States and southeastern Canada, including the island of Newfoundland, and small areas of suitable habitat in southwestern Canada and the Aleutians (Fig. 6). The area with high predicted occurrence probability of *L. niger* in the New World includes the two sites where populations have actually established (which were not used in the modeling): Nova Scotia and Vancouver. Further areas with high occurrence probabilities are New England, Southern Ontario, the Great Lakes-region and the Northern Appalachians. Low occurrence probabilities were found for the central North American prairies as well as arctic, boreal, arid, subtropical and tropical regions (Fig. 6). Considering the highest occurrence probability range (0.8–1 on a 0–1 probability scale), the area of suitable habitats for *L. niger* is 4,547,537 km^2^ in Europe and 1,308,920 km^2^ in North America. For an intermediate to high occurrence probability range (0.5–1) we estimated 5,371,055 km^2^ in Europe and 3,054,283 km^2^ in North America, and for the widest probability range (0.2–1) we estimated 6,155,643 km^2^ of suitable areas in Europe and 6,889,745 km^2^ in North America (Fig. 6).

## Discussion

In this study, we discovered a new cryptic species among the Nearctic ants previously known as “*L. niger”* and show evidence for the introduction of Palearctic *L. niger* to North America. *L. ponderosae* sp. nov., is endemic and thus native to North America (Fig. 1). Previous publications mentioning “*L. niger*” collected in North America most likely refer to this species. Our genetic analyses also show that the relatively large sister clade of *L. ponderosae* sp. nov. (the clade including *L. niger, L. platythorax, L. japonicus, L. emarginatus, L. balearicus, L. grandis, L. cinereus,* the *L. alienus*-complex, *L. sakagamii, L. productus* and *L. hayashi* in Fig. 1) is absent from the Nearctic region. The exception is a few populations of Palearctic *L. niger* found in anthropogenic habitats in the Canadian cities of Vancouver and Halifax, sharing the predominant DNA-barcode of our sampled *L. niger* populations in the Palearctic (Fig. 2). We interpret these finding as clear evidence that *L. niger* is an introduced species in Canada and the New World. Finding the same COI-mitotype for ants living in two continents is rare and suggests recent or ongoing dispersal. Recent natural dispersal of ants between Eurasia and North America occurred only via the Beringian land bridge^33^. However, within the Old World, *L. niger* is restricted to the western and central Palearctic region and has not been confirmed from eastern Asia^26^. Therefore, recent natural dispersal of this species to the North American continent is unlikely, due to the lack of natural dispersal routes. In addition, *L. niger* thus far has only been found in Canada near cities (Supplementary Table S2), a distribution pattern that is typical for exotic species and suggests that it is human-mediated. The origin of the introduced populations of *L. niger* in Canada remain to be discovered. In addition to expanded specimen sampling, finding representatives in the Palearctic region of the COI-mitotype, found to be unique for Canada among the samples investigated here (h4 in Fig. 2), may help to narrow down the range of potential sources of *L. niger* introduction.

*Lasius ponderosae* sp. nov. forms an unusually diverse clade that can be delineated using both molecular and morphological methods. *L. ponderosae* sp. nov. exhibits much higher COI-divergence than the usual genetic variation found within most *Lasius* species (Fig. 2). The maximum uncorrected divergence of COI within this taxon was found to be 5.9%, and 15 mitotypes were detected in 28 sequences. Haplotype diversity was high (0.899) and so was nucleotide diversity (0.012). Representatives of two of the four main COI-lineages also differ in two nuclear genes: Wg (0.1–0.4%) and Top1 (0.1–0.3%). Also, morphological variation within *L. ponderosae* sp. nov. was observed (see ranges of morphometric variables in Table 1). It is therefore possible that *L. ponderosae* sp. nov. represents a complex of poorly understood species rather than a single species. Investigations involving more samples from the entire range of this group, a deeper genomic scan and detailed morphological investigation involving all castes may clarify this question in the future. Finally, a population from the area of coastal Massachusetts that was hypothesized to belong to *L. niger* or yet another, undescribed species^19^ could previously not be distinguished from *L. neoniger* using DNA-barcoding^33^.

The introduction of *L. niger* to North America could potentially result in a serious biological invasion. *Lasius niger* was found in eleven separate outdoor localities (Supplementary Table S2). One specimen from Halifax imaged on AntWeb (casent0280452) displaying the morphological traits of *L. niger* was already collected in 1996, suggesting that *L. niger* workers could have been present in this area for at least 26 years. Finally, environmental niche modelling predicts highly suitable conditions in both Canadian sites of introduction (Fig. 3). We therefore conclude that *L. niger* is permanently established in Canada. Our samples from distant locations throughout the native European range and the introduced range in Canada formed a dense cluster of COI-mitotypes, only differing from each other in pairwise comparisons by a maximum of four nucleotide substitutions per 658 bp (0.6%), despite the vast population size of this most common European ant (Fig. 2a). Moreover, most specimens analyzed here belong to the same widespread European mitotype (Fig. 2a,b). Possible explanations of this genetic structure include (1) a young evolutionary age of the species followed by a rapid population expansion, (2) one or several bottlenecks during glaciation and subsequent rapid dispersal or (3) a selective sweep or infection with *Wolbachia* endosymbiont bacteria^54^. Among these possibilities, the first explanation seems the most likely as a recent analysis of the genome of this species revealed, *L. niger* apparently possesses genomic exaptations to urbanization^30^. *Lasius niger* is particularly common in gardens, cities and rural landscapes in Europe, but nearly absent from closed forests^55^, possibly representing large parts of the original European vegetation. Therefore, this species has benefitted strongly from anthropogenic land change which mediated its recent population expansion. The future impact of *L. niger* populations in North America is difficult to predict given our current information. Our niche modeling suggests a relatively large area of suitable habitat for the species in North America, but competition with native species may affect (limit) its dispersal in unexpected ways. Furthermore, on the one hand, some biological properties of *L. niger* seemingly limit its potential to cause ecological damage in natural habitats: (1) although *L. niger* queens may facultatively aggregate during colony foundation (pleometrosis), mature colonies are monogynous, i.e. headed by a single queen only^56^; (2) despite high ecological potential, *L. niger* is not known to produce supercolonial populations (colonies consisting of several connected nests); (3) the species shows intraspecific aggression at individual level^57^. On the other hand, even though *L. niger* is monogynous, the species has managed to reach an enormous abundance over a wide area within its native range, and information about its ecology is limited in its introduced range. As a true omnivore^55^, *L. niger* has the potential to strongly compete with native Nearctic ants possessing similar ecological niches and competitively displace them particularly in modified habitats. Laboratory aggression tests^58^ between *L. niger* and local native species would be a simple first step to explore these ecological interactions. In addition to displacing native ant species, *L. niger* may also negatively affect other native animals via direct predation^59^, or enhancement of predation risk^60^. Plants may be affected via the assistance of associated aphid species^61^. This could be the main economic damage caused by this species, in addition to minor structural damage in cities^62^. Finally, if left uncontrolled and over generations of adaptation to the novel range, *L. niger* may even expand its distribution into undisturbed natural habitats in North America, ultimately to the detriment of native biodiversity. We therefore propose (1) that the extent of the already established populations of *L. niger* in North America and their impact on local ecosystems should be closely monitored and studied; (2) that active control measures should be rapidly implemented to prevent already established populations from further spread. Our results underscore the necessity of integrating molecular phylogenetics, phylogeography and taxonomy in the timely recognition of non-native species introductions involving cryptic species.

## Supplementary Information

Supplementary Information.

## Data Availability

All primary data are accessible through the electronic supplementary material (Table S1-S6, Fig. S1), BOLD and GenBank. Voucher specimens are deposited at the Museum of Comparative Zoology, Cambridge, USA and at the Institut de Biologia Evolutiva (CSIC-UPF), Barcelona, Spain.
