# Supplementary material for: Integrative taxonomy reveals cryptic diversity in North American Lasius ants, and an overlooked introduced species
Source: Sci Rep. 2022 Apr 8;12:5970. doi: 10.1038/s41598-022-10047-9 (PMC8993915; doi:10.1038/s41598-022-10047-9)
Supplement: Supplementary file 1 — Supplementary Information. [file 41598_2022_10047_MOESM1_ESM.pdf]

Supplementary Material to "Integrative taxonomy reveals cryptic diversity in North American *Lasius* ants, and an overlooked introduced species"

Sämi Schär, Gerard Talavera, Jignasha D. Rana, Xavier Espadaler, Stefan P. Cover, Steven O. Shattuck and Roger Vila

Table S1. GenBank accession numbers for sequences used in the phylogenetic analysis. The sequences originate from the following papers: ref. 1 (Accession numbers starting with “LT”), ref. 2 (Accession numbers starting with “KC”), ref. 3 (Accession numbers starting with “AY”), ref. 4 (Accession numbers starting with “AB”), ref. 5 (Accession numbers starting with “EU”), ref. 6 (Accession number starting with “DQ”), ref. 7 (Accession number starting with “MF”), and new sequences from this study (Accession number starting with “OV”).

***Lasius* species**	**COI**	**COII**	**16S**	**Defensin**	**H3**	**LR**	**Wg**	**28S**	**Top1**
alienus	LT977438, AY225865	AY452152	KC820315		OV307173	KC820380	LT977308	LT977135	LT977225
americanus	LT977437						LT977292	LT977119	LT977250
austriacus	AY225873		AB371009, AB371055	EU401747					
balearicus	KC820337		KC820302		OV307178	KC820372	KC820396		
bombycina	LT977442						LT977297	LT977124	LT977220
brunneus	LT977443, OV307180	AY452153	KC820313		OV307175	KC820389	KC820411		
cinereus	KC820328		KC820287		OV307179	KC820362			
crypticus	LT977445						LT977317	LT977144	LT977230
emarginatus	LT977448, KC820359	AY452154	KC820323	EU401742	OV307177	KC820383	KC820391		
grandis	LT977473, KC820325		KC820293		OV307176	KC820379	KC820402		
hayashi	AB371013, AB371059		AB371013, AB371059						
japonicus	AB371015, AB371061		AB371015, AB371061	EU401745					
lasioides	LT977475, KC820347		KC820310	EU401748	OV307174	KC820386	KC820404		
mixtus	LT977482, AB370988, AB371034		AB370988, AB371034				LT977303	LT977130	LT977238
neglectus	LT977489		AB371018, AB371064						
neoniger	LT977492						LT977327	LT977154	LT977231
niger	LT977520, KC820356	MF993323	KC820320	EU401743	KC820367	KC820406	LT977282	LT977109	LT977223

pallitarsis	LT977529, AB371025	AY452156	AB371071						
paralienus	LT977531						LT977325	LT977152	LT977218
piliferus	LT977533						LT977288	LT977115	LT977226
platythorax	LT977539, AY225867		KC820318			KC820385	LT977272	LT977099	LT977221
ponderosae	LT977439						LT977278	LT977105	LT977222
ponderosae	LT977523						LT977313	LT977140	LT977227
ponderosae	LT977521						LT977314	LT977141	LT977219
ponderosae	LT977522						LT977323	LT977150	LT977224
productus	AB371021, AB371067		AB371021, AB371067						
psammophilus	LT977544, AY225863	AY452157	KC820322			KC820382	LT977273	LT977100	LT977228
sakagamii	AY225864		AB371022, AB371068	EU401744					
sitiens	LT977548								
turcicus	LT977559, DQ975428								
xerophilus	LT977582						LT977293	LT977120	LT977229

Table S2. GenBank accession numbers, BOLD identifiers, species name, locality of collection, COI mitotype and reference of specimens included in DNA-barcoding.

BOLD identifier	GenBank Accession No.	*Lasius* species	Country	Latitude	Longitude	Mitotype	Locality	Province	Ref.
BBFOB244-10	JN292013	*ponderosae* sp. nov.	Canada	52.92	-118.00	h8	Jasper National Park	Alberta	8
BBFOB302-10	JN292068	*ponderosae* sp. nov.	Canada	52.92	-118.00	h8	Jasper National Park	Alberta	8
BBFOB054-10	JF864360	*ponderosae* sp. nov.	Canada	52.92	-118.00	h9	Jasper National Park	Alberta	8
BBFOB122-10	JN291900	*ponderosae* sp. nov.	Canada	52.67	-117.88	h8	Jasper National Park	Alberta	8
BBFOB146-10	JN291922	*ponderosae* sp. nov.	Canada	52.67	-117.88	h8	Jasper National Park	Alberta	8
BBFOB175-10	JN291950	*ponderosae* sp. nov.	Canada	52.67	-117.88	h8	Jasper National Park	Alberta	8
BBFOB208-10	JN291980	*ponderosae* sp. nov.	Canada	52.92	-118.00	h9	Jasper National Park	Alberta	8
BBFOB357-10	JN292120	*ponderosae* sp. nov.	Canada	52.92	-118.00	h9	Jasper National Park	Alberta	8
BBFOB349-10	JN292113	*ponderosae* sp. nov.	Canada	52.67	-117.88	h8	Jasper National Park	Alberta	8
BBFOB367-10	JN292129	*ponderosae* sp. nov.	Canada	52.67	-117.88	h8	Jasper National Park	Alberta	8
BBFOB368-10	JN292130	*ponderosae* sp. nov.	Canada	52.67	-117.88	h8	Jasper National Park	Alberta	8
ANTBG078-10		*niger*	Bulgaria	42.68	23.35	h1	Sofia	Sofia	8
HPPPD197-13	KR785174	*niger*	Canada	44.62	-63.57	h2	Point Pleasant Park	Nova Scotia	8
HPPPE410-13		*niger*	Canada	44.62	-63.57	h2	Point Pleasant Park	Nova Scotia	8
HPPPE317-13	KR803473	*niger*	Canada	44.62	-63.57	h2	Point Pleasant Park	Nova Scotia	8
HPPPH364-13	KR807345	*niger*	Canada	44.62	-63.57	h2	Point Pleasant Park	Nova Scotia	8
HPPPI634-13	KR793992	*niger*	Canada	44.62	-63.57	h2	Point Pleasant Park	Nova Scotia	8
HPPPN468-13	KR794093	*niger*	Canada	44.62	-63.57	h2	Point Pleasant Park	Nova Scotia	8
SMTPP1600-15		*niger*	Canada	49.17	-122.64	h2	James Kennedy Elementary	British Columbia	8
SMTPP054-15		*niger*	Canada	49.32	-123.05	h4	Brooksbank Elementary School	British Columbia	8
SMTPO10842-15		*niger*	Canada	49.33	-123.13	h2	Ecole Cedardale	British Columbia	8
SMTPP1056-15		*niger*	Canada	45.37	-63.28	h2	Cobequid Educational Centre	Nova Scotia	8
SMTPP3952-15		*niger*	Canada	49.22	-122.61	h2	Maple Ridge Secondary	British Columbia	8

BOLD identifier	GenBank Accession No.	*Lasius* species	Country	Latitude	Longitude	Mitotype	Locality	Province	Ref.
SMTPP3790-15		*niger*	Canada	49.22	-122.61	h4	Maple Ridge Secondary	British Columbia	8
SMTPP3791-15		*niger*	Canada	49.22	-122.61	h2	Maple Ridge Secondary	British Columbia	8
SMTPP3792-15		*niger*	Canada	49.22	-122.61	h2	Maple Ridge Secondary	British Columbia	8
GMGRF5466-13		*niger*	Germany	48.95	13.42	h2	Nationalpark Bayerischer Wald	Bavaria	8
GMGRF3307-13		*niger*	Germany	48.95	13.42	h2	Nationalpark Bayerischer Wald	Bavaria	8
GMGRF3308-13		*niger*	Germany	48.95	13.42	h2	Nationalpark Bayerischer Wald	Bavaria	8
GMGRF6306-13		*niger*	Germany	48.95	13.42	h2	Nationalpark Bayerischer Wald	Bavaria	8
GMGRI682-13		*niger*	Germany	48.95	13.42	h2	Nationalpark Bayerischer Wald	Bavaria	8
SMTPL7018-15		*niger*	Canada	45.05	-64.74	h2	Berwick and District School	Nova Scotia	8
SMTPL6837-15		*niger*	Canada	45.05	-64.74	h2	Berwick and District School	Nova Scotia	8
SMTPL9849-15		*niger*	Canada	49.24	-122.78	h2	Citadel Middle School	British Columbia	8
SMTPL9862-15		*niger*	Canada	49.24	-122.78	h2	Citadel Middle School	British Columbia	8
SMTPL9873-15		*niger*	Canada	49.24	-122.78	h2	Citadel Middle School	British Columbia	8
SMTPL9880-15		*niger*	Canada	49.24	-122.78	h2	Citadel Middle School	British Columbia	8
SMTPL9884-15		*niger*	Canada	49.24	-122.78	h2	Citadel Middle School	British Columbia	8
SMTPL9891-15		*niger*	Canada	49.24	-122.78	h2	Citadel Middle School	British Columbia	8
SMTPL9847-15		*niger*	Canada	49.24	-122.78	h2	Citadel Middle School	British Columbia	8
SMTPL9857-15		*niger*	Canada	49.24	-122.78	h2	Citadel Middle School	British Columbia	8
SMTPL9859-15		*niger*	Canada	49.24	-122.78	h2	Citadel Middle School	British Columbia	8
SMTPL9865-15		*niger*	Canada	49.24	-122.78	h2	Citadel Middle School	British Columbia	8
SMTPL9869-15		*niger*	Canada	49.24	-122.78	h2	Citadel Middle School	British Columbia	8
SMTPL9888-15		*niger*	Canada	49.24	-122.78	h2	Citadel Middle School	British Columbia	8
SMTPL9892-15		*niger*	Canada	49.24	-122.78	h2	Citadel Middle School	British Columbia	8
SMTPL9894-15		*niger*	Canada	49.24	-122.78	h2	Citadel Middle School	British Columbia	8
SMTPL9861-15		*niger*	Canada	49.24	-122.78	h2	Citadel Middle School	British Columbia	8

BOLD identifier	GenBank Accession No.	*Lasius* species	Country	Latitude	Longitude	Mitotype	Locality	Province	Ref.
SMTPL9866-15		*niger*	Canada	49.24	-122.78	h4	Citadel Middle School	British Columbia	8
SMTPL9871-15		*niger*	Canada	49.24	-122.78	h2	Citadel Middle School	British Columbia	8
SMTPL9874-15		*niger*	Canada	49.24	-122.78	h2	Citadel Middle School	British Columbia	8
SMTPJ4652-14	KR876526	*niger*	Canada	49.25	-123.14	h2	Little Flower Academy	British Columbia	8
SMTPM2961-15		*niger*	Canada	49.22	-123.02	h2	Suncrest Elementary	British Columbia	8
SMTPM2964-15		*niger*	Canada	49.22	-123.02	h2	Suncrest Elementary	British Columbia	8
SMTPM2971-15		*niger*	Canada	49.22	-123.02	h2	Suncrest Elementary	British Columbia	8
SMTPM3004-15		*niger*	Canada	49.22	-123.02	h2	Suncrest Elementary	British Columbia	8
SMTPM3184-15		*niger*	Canada	49.22	-123.02	h2	Suncrest Elementary	British Columbia	8
SMTPM1557-15		*niger*	Canada	49.25	-122.90	h2	Cameron Elementary School	British Columbia	8
SMTPM1555-15		*niger*	Canada	49.25	-122.90	h2	Cameron Elementary School	British Columbia	8
SMTPM1556-15		*niger*	Canada	49.25	-122.90	h2	Cameron Elementary School	British Columbia	8
SMTPM1558-15		*niger*	Canada	49.25	-122.90	h2	Burnaby, Cameron Elementary School	British Columbia	8
SMTPM2984-15		*niger*	Canada	49.22	-123.02	h2	Burnaby, Suncrest Elementary	British Columbia	8
	KC820354	*niger*	Spain	41.50	-5.74	h1	Zamora	Castile and Léon	2
	KC820355	*niger*	Spain	42.11	2.21	h1	Montesquiu	Catalonia	2
	KC820356	*niger*	Germany	49.20	8.12	h3	Landau	Rhineland-Palatinate	2
	LT977522	*ponderosae* sp. nov.	USA	36.61	-112.35	h10	Kaibab Plateau	Arizona	1
	LT977523	*ponderosae* sp. nov.	USA	37.83	-109.51	h11	Abajo Mountains	Utah	1
	LT977521	*ponderosae* sp. nov.	USA	36.26	-115.64	h12	Spring Mountains	Nevada	1
	LT977520	*niger*	Denmark	56.46	10.04	h2	Randers	Midtjylland	1
	LT977510	*niger*	Russia	55.12	37.69	h2	Bershovo	Moscow	1
	LT977509	*niger*	Switzerland	46.13	7.63	h2	Zinal	Valais	1
	LT977501	*niger*	Russia	50.16	86.31	h6	Tyungur	Altai	1
	LT977502	*niger*	Russia	53.40	91.17	h2	Alshanovo	Khakassia	1

BOLD identifier	GenBank Accession No.	*Lasius* species	Country	Latitude	Longitude	Mitotype	Locality	Province	Ref.
	LT977499	*niger*	Estonia	58.92	26.28	h6	Seljajärv	Lääne-Virumaa	1
	LT977498	*niger*	Estonia	59.34	25.69	h2	Jussi	Harjumaa	1
	LT977519	*ponderosae* sp. nov.	USA	36.61	-112.35	h13	Kaibab Plateau	Arizona	1
	LT977514	*ponderosae* sp. nov.	USA	36.68	-112.22	h14	Kaibab Plateau	Arizona	1
	LT977518	*ponderosae* sp. nov.	USA	39.07	-108.09	h15	12.6 miles southeast of junction Route 330 on Route 65	Colorado	1
	LT977516	*ponderosae* sp. nov.	USA	38.37	-109.17	h15	La Sal Mountains	Utah	1
	LT977494	*ponderosae* sp. nov.	USA	38.51	-109.32	h11	La Sal Mountains	Utah	1
	LT977517	*ponderosae* sp. nov.	USA	36.74	-112.22	h16	Kaibab Plateau	Arizona	1
	LT977513	*ponderosae* sp. nov.	USA	37.44	-112.53	h17	Stout Canyon	Utah	1
	LT977512	*ponderosae* sp. nov.	USA	36.31	-115.61	h18	Spring Mountains	Nevada	1
	LT977515	*ponderosae* sp. nov.	USA	39.09	-111.31	h17	Ferron Mountain	Utah	1
	LT977508	*ponderosae* sp. nov.	USA	40.66	-109.48	h17	Uinta Mountains	Utah	1
	LT977507	*ponderosae* sp. nov.	USA	40.70	-109.49	h19	Uintah	Utah	1
	LT977506	*niger*	Switzerland	47.39	8.55	h5	Zurich	Zurich	1
	LT977504	*niger*	Sweden	58.89	14.01	h2	Vänern, E shore	Västra Götaland	1
	LT977503	*niger*	Sweden	55.70	13.47	h2	Krankesjön	Skåne	1
	LT977500	*niger*	Finland	60.45	22.29	h2	Turku	Southwest Finland	1
	LT977495	*niger*	Bulgaria	43.36	28.08	h3	Albena	Dobrich	1
	LT977505	*niger*	Switzerland	46.21	6.15	h2	Geneva	Geneva	1
	LT977497	*niger*	Croatia	43.81	15.96	h2	Krka National Park	Šibensko-Kninska	1
	LT977496	*niger*	Bulgaria	42.71	23.32	h7	Sofia	Sofia	1
	LT977511	*niger*	Russia	55.96	37.40	h6	Moscow	Moscow	1
	LT977549	*ponderosae* sp. nov.	USA	36.34	-115.65	h20	Spring Mountains	Nevada	1

**BOLD identifier**	**GenBank Accession No.**	***Lasius* species**	**Country**	**Latitude**	**Longitude**	**Mitotype**	**Locality**	**Province**	**Ref.**
	LT977439	*ponderosae* sp. nov.	USA	31.92	-109.26	h21	Chiricahua Mountains	Arizona	1
	OV307181	*niger*	NA	NA	NA	h2	NA	NA	This study

NA: not available

Table S3. Definition of morphometric variables

CL	The maximum measurable distance from the midpoint of clypeus to the centre of the hind margin of the head capsule, in mm.
CW	Maximum width of head. This may include the compound eyes, if those mark the outmost point. Measured in mm.
CS	Mean of CL and CW, in mm.
dCLAN	Torulo-clypeal distance. The minimal distance between the inner margin of the antennal socket and the posterior marginal clypeal suture, in mm.
EYE	Eye size. The mean between the maximal length of the compound eye (EL) and its minimum diameter (EW), in mm.
GuHL	Length of longest hair on underside of head, in mm.
MaDe	Unilateral number of mandibular teeth.
MP6	Length of the 6th (terminal) segment of the maxillary palps.
nGen	In perfect full face view (dorsal view on head), unilateral number of standing hairs (setae) protruding ≥20µm from the genae, i.e. from the anterior margin of the compound eye to the lateral posterior margin of the clypeus.
nGu	Unilateral number of long, standing hairs (setae) protruding ≥20µm from the underside of head.
nHT	With large diameter of hind tibia in visual plane, unilateral number of long, standing hairs (setae) protruding ≥20µm from the extensor (outer) side of the hind tibia.
nOcc	In perfect full face view, unilateral number of long hairs (setae) protruding ≥20µm from the posterior margin of the head. From the midpoint of the posterior margin of the head to the level of the posterior end of the compound eye.
nSc	With small diameter of antennal scape in visual plane, unilateral number of long, standing hairs (setae) protruding ≥20µm from the dorsal surface of the antennal scape.
nSt	Unilateral number of ≥20µm long, standing hairs (setae) on the lower sides of the propodeum. Those hairs are best visible with the ant in lateral view, and a light source shining on the specimen in horizontal (front to back) direction. The area of hair count starts below the level of the propodeal stigma, excluding the area adjacent to the metapleural gland and setae along the ventral edge of the mesosoma. Transition area between lateral and posterior slope of propodeum included.
PLF	Mean length of microscopic, adjacent pubescence hairs on the forehead, between the frontal lobes. Only pubescence hairs for which both endpoints are clearly visible should be measured, but among those, hairs should be chosen at random. A method of avoiding selection bias is to start with one hair and move in one direction, measuring every sufficiently visible hair. At least 7 measurements should be taken, if possible.
PnHL	Length of the longest hair on the dorsal pronotum.
PoOc	The fraction of CL starting posterior of the level of the compound eye. In case of head asymmetry, the mean of left and right postocular distance should be calculated.

SL	Maximum straight line length of antennal scape, excluding the joint attached to the head capsule.
sqPDCL	Square root of mean pubescence distance on the lateral clypeus, in µm. The number of adjacent pubescence hairs crossing a virtual line across one side of the clypeus is counted in perpendicular view. This line starts from the midpoint of the posterior margin of the clypeus to one of the two midpoints of the lateral clypeal depressions. The length of the virtual line is then divided by the number of pubescence hairs counted. Finally, the square root is calculated.

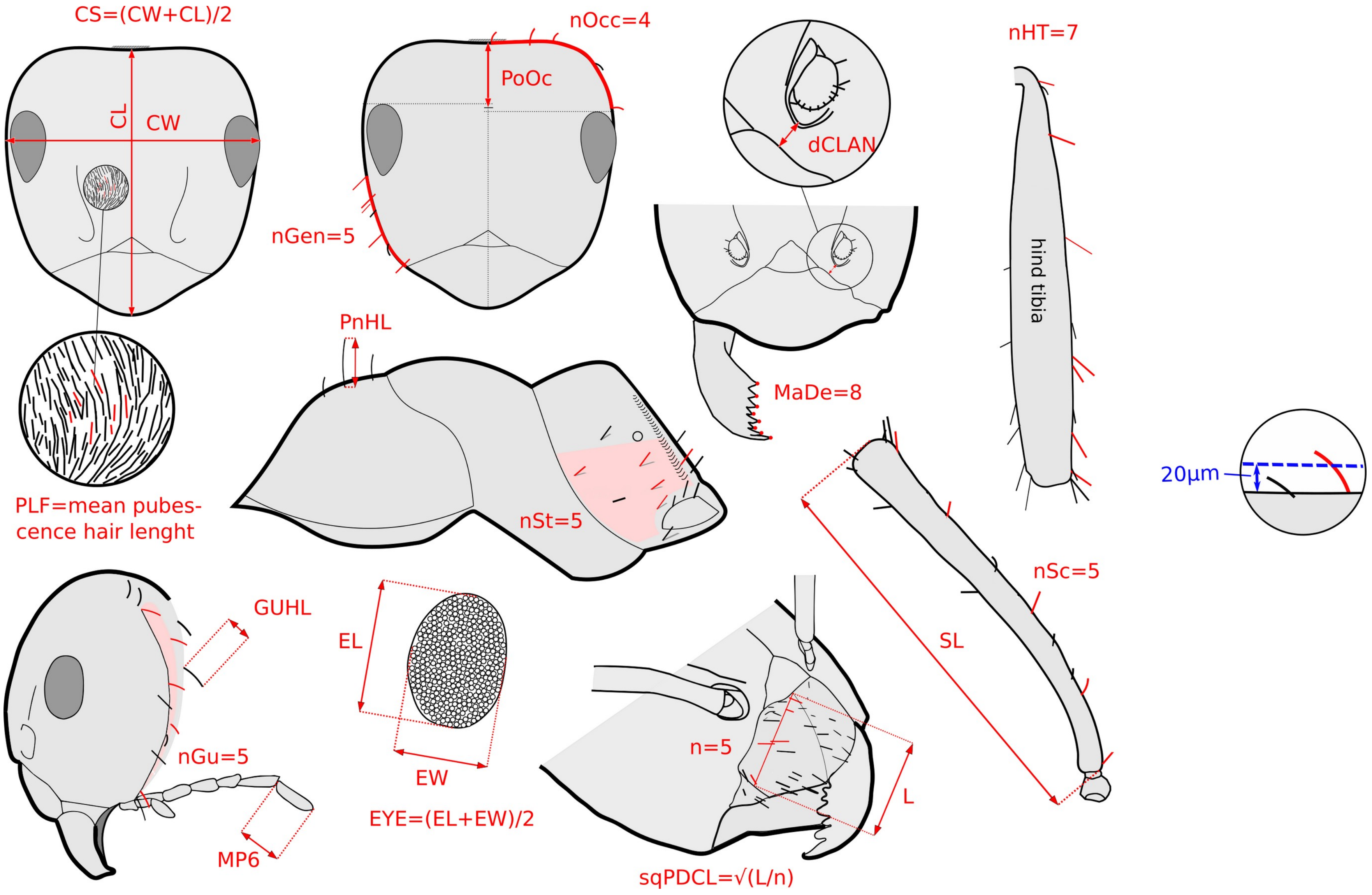

Fig S1. Definition of morphometric variables.

Table S4. Absence and presence data underlying the ecological niche modeling carried out in this study and references for each record.

Latitude	Longitude	Data type	Reference
27.97	-15.61	absence	9
28.90	-10.17	absence	10
29.27	-9.73	absence	10
29.69	-9.73	absence	10
29.79	-8.78	absence	10
30.42	-9.60	absence	10
30.49	-8.85	absence	10
31.00	13.00	absence	10
31.00	22.00	absence	10
33.61	-7.63	absence	10
35.20	24.90	absence	11
36.72	-4.40	absence	10
37.10	-3.55	absence	10
37.18	-3.59	absence	10
37.39	-6.00	absence	10
37.55	-2.61	absence	10
38.05	14.78	absence	11
38.06	-1.09	absence	10
38.28	-0.83	absence	10
38.41	14.95	absence	11
38.70	-9.42	absence	10
39.00	-0.26	absence	10
39.04	-0.54	absence	10
39.13	-0.37	absence	10

Latitude	Longitude	Data type	Reference
39.46	-0.32	absence	10
39.61	2.99	absence	10
39.86	-4.03	absence	10
40.10	0.01	absence	10
41.37	0.94	absence	10
41.39	-1.57	absence	10
41.43	2.17	absence	10
41.53	1.18	absence	10
41.55	0.65	absence	10
41.56	1.24	absence	10
41.59	1.83	absence	10
41.61	1.19	absence	10
41.64	-0.90	absence	10
41.67	2.00	absence	10
41.75	2.12	absence	10
41.77	12.23	absence	S. Schär, personal observation
41.79	2.34	absence10	10
42.09	12.27	absence	S. Schär, personal observation
61.00	12.00	absence	12
63.00	13.80	absence	12
64.50	12.82	absence	12
64.70	14.50	absence	12
66.19	24.00	absence	12
67.04	23.40	absence	12
67.32	21.09	absence	12
67.88	18.90	absence	12

Latitude	Longitude	Data type	Reference
68.00	36.00	absence	9
68.29	18.93	absence	12
69.94	23.04	absence	12
43.12	11.80	absence	S. Schär, personal observation
39.60	22.09	absence	13
39.50	20.96	absence	13
37.60	22.20	absence	13
32.99	35.40	absence	14
34.45	36.16	absence	9
42.42	18.79	absence	15
42.20	18.96	absence	15
42.18	18.99	absence	15
42.22	19.02	absence	15
42.23	19.11	absence	15
42.22	19.12	absence	15
42.10	19.14	absence	15
42.40	19.38	absence	15
38.10	30.00	absence	10
39.30	33.80	absence	10
39.30	39.60	absence	10
37.50	28.00	absence	10
35.13	36.96	absence	10
31.14	-7.96	absence	10
31.50	-6.48	absence	10
40.40	9.10	absence	11
39.40	8.80	absence	11

**Latitude**	**Longitude**	**Data type**	**Reference**
37.80	13.10	absence	11
36.20	9.50	absence	10
64.00	10.88	absence	12
64.98	11.84	absence	12
65.84	12.89	absence	12
66.42	13.60	absence	12
67.00	14.65	absence	12
67.84	16.06	absence	12
68.66	15.89	absence	12
70.40	30.47	absence	12
65.56	21.81	absence	12
63.10	13.42	absence	12
62.04	14.41	absence	12
63.55	25.68	absence	12
39.07	29.69	absence	10
41.45	33.70	absence	10
39.55	39.75	absence	10
40.83	38.91	absence	10
64.34	39.50	absence	10
33.60	-3.93	absence	10
32.89	2.43	absence	10
32.41	21.45	absence	10
31.39	20.26	absence	10
34.52	36.48	absence	9
33.41	35.85	absence	9
27.7	-17.97	absence	9

Latitude	Longitude	Data type	Reference
28.69	-17.85	absence	9
28.26	-16.62	absence	9
64.91	-18.52	absence	12
65.63	-18.3	absence	12
65.47	-15.04	absence	12
62.68	38.26	absence	9
64.88	11.18	absence	12
66.51	13.11	absence	12
37.98	-1.08	presence	16
40.46	-3.67	presence	16
41.16	-8.68	presence	10
41.51	-5.74	presence	2
42.11	2.21	presence	2
42.27	-8.67	presence	16
42.71	23.32	presence	16
43.19	-0.61	presence	16
43.28	-2.17	presence	M. Menchetti, pers. collection
43.33	-1.99	presence	16
43.36	28.08	presence	16
43.68	4.63	presence	16
43.81	15.96	presence	1
43.85	10.51	presence	16
43.85	3.52	presence	17
44.89	1.22	presence	17
45.69	5.91	presence	16
45.92	6.87	presence	16

Latitude	Longitude	Data type	Reference
46.13	7.63	presence	1
47.62	-0.08	presence	17
48.95	13.42	presence	8
49.20	8.12	presence	2
49.58	19.53	presence	16
50.06	-5.71	presence	16
50.94	6.96	presence	17
51.28	4.33	presence	17
51.42	16.70	presence	16
51.66	-0.64	presence	16
51.81	-1.31	presence	16
52.36	-1.17	presence	16
52.68	4.70	presence	16
53.40	-9.92	presence	16
53.55	21.02	presence	16
54.38	-2.91	presence	16
55.12	37.69	presence	1
55.49	12.06	presence	16
55.70	13.47	presence	16
55.96	37.41	presence	16
56.46	10.04	presence	16
56.86	-5.42	presence	17
58.25	22.51	presence	16
58.89	14.01	presence	16
58.92	26.28	presence	16
59.34	25.69	presence	16

Latitude	Longitude	Data type	Reference
59.36	17.84	presence	17
59.61	9.14	presence	16
60.13	18.64	presence	16
60.29	10.62	presence	16
60.45	22.29	presence	16
61.04	7.59	presence	16
46.89	23.14	presence	18
49.02	26.34	presence	19
53.43	14.55	presence	20
52.87	17.92	presence	20
53.78	19.08	presence	20
51.73	19.78	presence	20
52.88	18.79	presence	20
52.43	16.95	presence	20
52.14	20.72	presence	20
51.71	19.63	presence	20
52.51	23.24	presence	20
52.16	22.28	presence	20
50.22	19.83	presence	20
50.10	20.49	presence	20
51.40	21.97	presence	20
47.70	22.38	presence	21
40.42	29.72	presence	22
40.53	29.83	presence	22
40.55	30.12	presence	22
40.69	0.76	presence	10

Latitude	Longitude	Data type	Reference
35.44	4.6	absence	23
36.75	3.06	presence	24
41.54	23.42	presence	24
50.13	13.94	presence	24
50.26	14.97	presence	24
49.92	14.02	presence	24
49.82	14.94	presence	24
49.3	14.04	presence	24
49.15	14.69	presence	24
49.08	13.78	presence	24
50.22	13.61	presence	24
50.68	15.84	presence	24
50.62	15.77	presence	24
50.63	15.52	presence	24
50.23	13.68	presence	24
49.49	14.02	presence	24
49.76	14.62	presence	24
56.23	10.55	presence	24
56.23	10.55	presence	24
59.93	24.37	presence	24
47.35	0.84	presence	24
47.73	6.1	presence	24
46.06	6.61	presence	24
42.67	9.28	presence	24
47.77	6.28	presence	24
43.2	3.08	presence	24

Latitude	Longitude	Data type	Reference
57.63	-3.73	presence	24
50.46	2.45	presence	24
50.69	-2.11	presence	24
54.41	12.46	presence	24
51.19	10.6	presence	24
50.77	12.44	presence	24
50.77	12.43	presence	24
50.77	12.43	presence	24
50.77	12.43	presence	24
51.22	14.95	presence	24
51.16	14.99	presence	24
51.16	14.99	presence	24
51.16	14.99	presence	24
51.16	14.99	presence	24
51.16	14.99	presence	24
51.16	14.99	presence	24
51.16	14.99	presence	24
51.2	14.95	presence	24
51.2	14.95	presence	24
51.2	14.95	presence	24
51.2	14.95	presence	24
51.2	14.95	presence	24
51.2	14.95	presence	24
51.06	14.93	presence	24
51.2	14.85	presence	24
51.19	14.84	presence	24

Latitude	Longitude	Data type	Reference
51.19	14.84	presence	24
51.49	13.79	presence	24
51.33	12.39	presence	24
51.32	12.4	presence	24
51.35	12.41	presence	24
51.34	12.38	presence	24
51.28	14.57	presence	24
51.26	14.79	presence	24
51.26	14.79	presence	24
51.26	14.79	presence	24
51.26	14.79	presence	24
51.26	14.79	presence	24
51.26	14.79	presence	24
51.26	14.79	presence	24
50.19	6.45	presence	24
53.79	12.8	presence	24
51.2	14.37	presence	24
50.91	11.22	presence	24
50.68	12.46	presence	24
50.68	12.46	presence	24
41.77	13.91	presence	24
41.77	13.86	presence	24
50.15	19.79	presence	24
48.49	20.14	presence	24
48.49	20.14	presence	24
41.2	-8.7	presence	24

Latitude	Longitude	Data type	Reference
59.9	30.4	presence	24
48.23	18.67	presence	24
47.77	18.52	presence	24
48.37	18.65	presence	24
48.82	22.03	presence	24
48.55	17.52	presence	24
40.72	0.74	presence	24
40.43	-3.68	presence	24
42.59	1.07	presence	24
42.59	1.07	presence	24
56.02	14.83	presence	24
56.22	15.42	presence	24
59.82	17.78	presence	24
56.68	16.52	presence	24
57.23	17.05	presence	24
56.44	12.57	presence	24
55.43	14.18	presence	24
55.67	13.55	presence	24
38.22	37.25	presence	24
11.81	-15.19	absence	9
13.25	-16.39	absence	9
12.07	-12.29	absence	9
13.15	-10.23	absence	9
10.35	-14.33	absence	9
9.21	-11.12	absence	9
6.24	-9.99	absence	9

Latitude	Longitude	Data type	Reference
5.06	-7.58	absence	9
6.09	-5.88	absence	9
5.74	-4.04	absence	9
6.14	-0.1	absence	9
7.72	0.74	absence	9
6.96	4.92	absence	9
12.17	2.52	absence	9
13.21	14.1	absence	9
8.1	22.02	absence	9
2.88	25.14	absence	9
7.97	35.09	absence	9
13.54	37.22	absence	9
6.61	39.65	absence	9
7.99	35.14	absence	9
24.53	32.97	absence	9
24.74	24.31	absence	9
21.48	18	absence	9
56.46	10.04	presence	1
55.12	37.69	presence	1
46.13	7.63	presence	1
58.92	26.28	presence	1
59.34	25.69	presence	1
47.39	8.55	presence	1
58.89	14.01	presence	1
55.7	13.47	presence	1
60.45	22.29	presence	1

Latitude	Longitude	Data type	Reference
43.36	28.08	presence	1
46.21	6.15	presence	1
43.81	15.96	presence	1
42.71	23.32	presence	1
55.96	37.4	presence	1
29.52	2.16	absence	9
26.03	11.36	absence	9
23.22	-14.44	absence	9
23.03	-7.98	absence	9
21.93	-3.01	absence	9
26.06	7.95	absence	9
23.82	11.38	absence	9
30.99	30.96	absence	9
23.98	14.67	absence	9
19.97	18.18	absence	9
18.27	14.45	absence	9
18.73	32.23	absence	9
19.1	26.06	absence	9
11.54	20.89	absence	9
14.14	-9.8	absence	9
16.31	-1.04	absence	9
0.87	12.65	absence	9
2.33	17.51	absence	9
0.31	21.76	absence	9
2.4	37.73	absence	9
4.04	19.79	absence	9

Latitude	Longitude	Data type	Reference
25.73	-8.99	absence	9
24.9	-8.91	absence	9
24.55	-1.69	absence	9
16.2	-14.84	absence	9
15.18	-9.67	absence	9
19.49	4.53	absence	9
28.16	-4.05	absence	9
28.26	26.67	absence	9
30.18	33.8	absence	9
26.87	37.38	absence	9
29.34	38.53	absence	9
21.84	39.55	absence	9
23.97	35.04	absence	9
17.41	37.74	absence	9
27.69	17.68	absence	9
29.63	10.19	absence	9
25.26	-0.88	absence	9
16.32	0.88	absence	9
7.6	8.17	absence	9
10.58	13.76	absence	9
16.74	12.87	absence	9
1.41	26.53	absence	9
3.96	16.38	absence	9
7.03	5.1	absence	9
5.22	28.96	absence	9
0.3	30.03	absence	9

Table S5: Standard morphometric data of the holotype and paratypes of *Lasius ponderosae* sp. nov. Measurements are given in mm.

**Specimen**	**Holotype**	**W**	**W**	**W**	**W**	**W**	**M**	**M**	**Q**	**Q**
HL	0.899	0.864	0.812	0.848	0.857	0.826	0.659	0.673	1.446	1.42
HW	0.823	0.823	0.758	0.797	0.798	0.764	0.698	0.766	1.592	1.524
SL	0.821	0.802	0.768	0.782	0.781	0.781	0.506	0.519	1.207	1.197
EL	0.239	0.234	0.211	0.227	0.221	0.219	0.28	0.294	0.415	0.394
EW	0.189	0.182	0.173	0.194	0.169	0.167	0.21	0.229	0.3	0.3
ProW	0.56	0.553	0.522	0.538	0.53	0.52	0.821	0.871	2.051	1.933
ML	1.069	1.018	0.946	1.028	0.974	0.971	1.313	1.45	2.804	2.83
HTL	0.863	0.82	0.996	0.848	0.817	0.798	0.918	0.997	1.667	1.647
CI	92	95	93	94	93	92	106	114	110	107
SI	100	97	101	98	98	102	72	68	76	79

HL Head length. Measured in full-face view, from mid-point of anterior clypeal margin to mid-point of posterior margin.
HW Maximum head width, measured in full-face view posterior of the eyes.
SL Maximum straight-line scape length excluding the articular condyle.
EL Eye length. Measured along the maximum diameter of eye.
EW Eye width. Measured along the maximum horizontal diameter of eye.
ProW Maximal with of prontoum in dorsal view.
ML Mesosoma length. Measured as diagonal length from the anterior end of the neck shield to the posterior margin of the propodeal lobe.
HTL Hind tibia length. Maximum length of hind tibia.
CI HW/HL*100
SI SL/HW*100

Table S6. Morphometric data of 39 workers of *L. ponderosae* sp. nov. and 49 workers of Palearctic members of the *L. niger*-complex. Measurements are given in mm, except sqPDCL, given in µm.

CollectionCode	*Lasius* species	Lat/lon	Co-unt-ry	Ma-De	CW	CL	CS	SL	sq-PDCL	PoOc	n-Gen	EYE	n-GU	n-OCC	GU-HL	dCL-AN	MP6	Pn-HL	nSt	n-HT	n-SC	PLF
MMP01321	*ponderosae* sp. nov.	38.52'N, 121.76'W	US	8	0.889	0.953	0.921	0.869	3.903	0.244	11	0.212	14	17.5	0.081	0.026	0.150	0.107	10	16	29	0.038
MMP01321	*ponderosae* sp. nov.	38.52'N, 121.76'W	US	8	0.837	0.895	0.866	0.828	3.909	0.216	11	0.215	11	13.5	0.087	0.026	0.144	0.078	9	13	4.5	0.031
MMP01321	*ponderosae* sp. nov.	38.52'N, 121.76'W	US	8	0.722	0.808	0.765	0.725	4.219	0.195	10	0.180	12	15	0.066	0.020	0.121	0.087	10	11	14	0.026
MMP01355	*ponderosae* sp. nov.	33.98'N, 120.07'W	US	8	0.779	0.837	0.808	0.743	5.221	0.219	15	0.191	11	22	0.110	0.038	0.115	0.101	6	25	21	0.029
MMP01355	*ponderosae* sp. nov.	33.98'N, 120.07'W	US	NA	0.751	0.837	0.794	0.775	5.635	0.202	15	0.184	9	19	0.073	0.040	0.118	0.095	7	23	25	0.027
MMP01355	*ponderosae* sp. nov.	33.98'N, 120.07'W	US	8	0.716	0.794	0.755	0.693	4.684	0.192	15	0.179	10	19	0.104	0.039	0.105	0.118	7	25	20	0.037
MMP01374	*ponderosae* sp. nov.	33.99'N, 120.06'W	US	8	0.837	0.895	0.866	0.808	7.087	0.234	15	0.211	15	18.5	0.130	0.035	0.115	0.165	6	33	25	0.033
MMP01374	*ponderosae* sp. nov.	33.99'N, 120.06'W	US	8	0.707	0.808	0.758	0.736	5.561	0.191	13.5	0.182	13	18	0.087	0.029	0.127	0.098	7	29	23	0.037
MMP01374	*ponderosae* sp. nov.	33.99'N, 120.06'W	US	8	0.664	0.733	0.699	0.655	5.967	0.178	9	0.179	9	18.5	0.087	0.029	0.121	0.075	7	21	18	0.033
MMP01394	*ponderosae* sp. nov.	33.98'N, 120.08'W	US	NA	0.895	0.970	0.932	0.878	5.373	0.238	18	0.231	21	26	0.107	0.040	0.141	0.124	9	32	33	0.031
MMP01394	*ponderosae* sp. nov.	33.98'N, 120.08'W	US	8	0.817	0.875	0.846	0.808	5.699	0.205	14.5	0.205	12	16	0.110	0.043	0.144	0.115	6	31	18	0.033
MMP01394	*ponderosae* sp. nov.	33.98'N, 120.08'W	US	NA	0.808	0.880	0.844	0.808	5.221	0.216	14	0.202	14	24	0.095	0.040	0.136	0.113	9	30	23	0.039
MMP02369	*ponderosae* sp. nov.	36.31'N, 115.62'W	US	7	0.817	0.875	0.846	0.837	6.092	0.221	12	0.188	23	22	0.115	0.038	0.130	0.121	8	33	39	0.034
MMP02369	*ponderosae* sp. nov.	36.31'N, 115.62'W	US	8	0.808	0.886	0.847	0.837	6.092	0.219	17	0.189	14	19	0.107	0.038	0.144	0.095	8	26	33	0.035
MMP02369	*ponderosae* sp. nov.	36.31'N, 115.62'W	US	7	0.779	0.866	0.823	0.808	6.922	0.202	15	0.192	14	17	0.121	0.038	0.127	0.095	6	24	30	0.028
MMP02410	*ponderosae* sp. nov.	36.28'N, 115.63'W	US	8	0.837	0.886	0.862	0.837	7.208	0.188	11.5	0.206	24	15	0.107	0.043	0.139	0.107	6	34	26	0.033
MMP02410	*ponderosae* sp. nov.	36.28'N, 115.63'W	US	8	0.895	0.924	0.909	0.895	7.208	0.225	18	0.209	23	20	0.115	0.046	0.165	0.118	7	32	31	0.038
MMP02410	*ponderosae* sp. nov.	36.28'N, 115.63'W	US	8	0.794	0.857	0.826	0.808	5.578	0.202	16	0.196	13	16.5	0.095	0.035	0.144	0.115	5	23	25	0.035
MMP02414	*ponderosae* sp. nov.	36.27'N, 115.63'W	US	8	0.779	0.866	0.823	0.808	6.204	0.202	12	0.191	12	18	0.107	0.046	0.133	0.118	7	26	28	0.035
MMP02414	*ponderosae* sp. nov.	36.27'N, 115.63'W	US	NA	0.800	0.872	0.836	0.817	5.011	0.202	11	0.192	13	19	0.113	0.043	0.144	0.118	9	30	14	0.027
MMP02414	*ponderosae* sp. nov.	36.27'N, 115.63'W	US	8	0.808	0.880	0.844	0.837	5.282	0.221	19	0.193	19	24	0.095	0.038	0.150	0.118	6	35	35	0.035
MMP02419	*ponderosae* sp. nov.	36.26'N, 115.63'W	US	8	0.878	0.924	0.901	0.892	6.092	0.219	13	0.211	23	17	0.118	0.043	0.144	0.141	6	25	33	0.030
MMP02419	*ponderosae* sp. nov.	36.26'N, 115.63'W	US	8	0.895	0.953	0.924	0.904	5.373	0.222	12	0.204	17	21	0.104	0.043	0.144	0.113	7	32	26	0.034
MMP02419	*ponderosae* sp. nov.	36.26'N, 115.63'W	US	8	0.878	0.938	0.908	0.880	6.092	0.231	9	0.211	11	19	0.110	0.042	0.144	0.124	8	31	32	0.044
MMP02773	*ponderosae* sp. nov.	38.18'N, 119.19'W	US	8	0.826	0.860	0.843	0.794	5.467	0.208	13	0.196	9	18	0.095	0.035	0.124	0.121	8	26	24	0.033

CollectionCode	*Lasius* species	Lat/lon	Co-unt-ry	Ma-De	CW	CL	CS	SL	sq-PDCL	PoOc	n-Gen	EYE	n-GU	n-OCC	GU-HL	dCL-AN	MP6	Pn-HL	nSt	n-HT	n-SC	PLF
MMP02773	*ponderosae* sp. nov.	38.18'N, 119.19'W	US	8	0.837	0.889	0.863	0.802	5.181	0.206	14	0.206	16	15	0.095	0.032	0.136	0.110	8	32	25	0.033
MMP02773	*ponderosae* sp. nov.	38.18'N, 119.19'W	US	NA	0.779	0.846	0.813	0.802	6.624	0.202	13	0.201	9	19	0.058	0.035	0.136	0.118	6	25	24	0.028
MMP02993	*ponderosae* sp. nov.	38.58'N, 119.33'W	US	8	0.866	0.906	0.886	0.837	6.007	0.211	12	0.220	23	21	0.124	0.043	0.133	0.124	9	35	31	0.036
MMP02993	*ponderosae* sp. nov.	38.58'N, 119.33'W	US	8	0.866	0.915	0.891	0.901	5.373	0.202	18	0.215	18	20	0.115	0.049	0.144	0.127	7	31	30	0.035
MMP02993	*ponderosae* sp. nov.	38.58'N, 119.33'W	US	8	0.779	0.820	0.800	0.779	4.953	0.185	11	0.202	19	20	0.095	0.038	0.130	0.107	7	28	27	0.032
MMP02994	*ponderosae* sp. nov.	38.58'N, 119.33'W	US	8	0.751	0.808	0.779	0.774	5.699	0.193	7	0.188	11	16	0.098	0.035	0.136	0.124	11	25	23	0.032
MMP02994	*ponderosae* sp. nov.	38.58'N, 119.33'W	US	NA	0.693	0.765	0.729	0.727	4.738	0.185	12	0.189	11	13	0.066	0.032	0.115	0.087	6	23	21	0.028
MMP02994	*ponderosae* sp. nov.	38.58'N, 119.33'W	US	NA	0.687	0.742	0.714	0.716	5.002	0.176	12	0.183	10	13	0.058	0.023	0.141	0.118	10	24	19	0.028
SPC8028	*ponderosae* sp. nov.	36.34'N, 115.65'W	US	7	0.808	0.878	0.843	0.837	5.097	0.225	13	0.202	23	24	0.078	0.038	0.139	0.127	7	26	22	0.040
SPC8028	*ponderosae* sp. nov.	36.34'N, 115.65'W	US	7	0.904	0.973	0.938	0.888	7.367	0.237	14	0.213	13	19	0.098	0.040	0.153	0.127	7	21	24	0.044
SPC8028	*ponderosae* sp. nov.	36.34'N, 115.65'W	US	7	0.831	0.889	0.860	0.852	5.603	0.216	13	0.202	13	18	0.107	0.029	0.150	0.144	8	27	21	0.048
SPC8571*	*ponderosae* sp. nov.	40.65'N, 109.46'W	US	8	0.895	0.924	0.909	0.883	5.097	0.208	10	0.232	8	16	0.107	0.040	0.144	0.115	6	27	22	0.038
SPC8571*	*ponderosae* sp. nov.	40.65'N, 109.46'W	US	7	0.886	0.909	0.898	0.866	5.097	0.231	11	0.216	7	17	0.098	0.046	0.141	0.118	5	29	26	0.042
SPC8590	*ponderosae* sp. nov.	40.7'N, 109.48'W	US	8	0.751	0.820	0.785	0.808	4.387	0.202	5	0.193	4	9	0.072	0.020	0.162	0.098	4	14	3.5	0.038
SS20B852	*cinereus*	44.77'N, 6.58'E	FR	8	0.794	0.875	0.834	0.870	4.482	0.196	10	0.202	11	17	0.072	0.040	0.182	0.127	3	18	25	0.025
SS20B852	*cinereus*	44.77'N, 6.58'E	FR	NA	0.823	0.875	0.849	0.866	3.670	0.196	5	0.201	7.5	11.5	0.087	0.049	0.144	0.123	3	19	23	0.023
SS16A960	*cinereus*	41.38'N, 0.96'E	ES	8	0.693	0.771	0.732	0.751	5.576	0.191	5	0.178	5	9.5	0.061	0.038	0.157	0.095	3	16	15	0.028
SS16A960	*cinereus*	41.38'N, 0.96'E	ES	NA	0.722	0.808	0.765	0.808	5.686	0.195	4	0.175	3	9.5	0.064	0.040	0.153	0.098	2	13	13	0.027
SS16A960	*cinereus*	41.38'N, 0.96'E	ES	8	0.664	0.751	0.707	0.742	5.744	0.178	3	0.175	2	8	0.064	0.040	0.162	0.089	2	10	16	0.027
RVcoll17S374	*grandis*	36.72'N, 4.41'W	ES	8	1.085	1.149	1.117	1.097	4.601	0.280	15.5	0.254	16	24.5	0.110	0.058	0.222	0.130	9	37	26	0.027
RVcoll17S374	*grandis*	36.72'N, 4.41'W	ES	8	1.082	1.117	1.100	1.097	4.663	0.260	14	0.260	22	24	0.098	0.058	0.202	0.147	10	32	30	0.031
RVcoll17S374	*grandis*	36.72'N, 4.41'W	ES	8	1.010	1.068	1.039	0.981	4.942	0.260	14	0.260	17	16.5	0.101	0.049	0.202	0.133	6	28	27	0.028
RVcoll16B133	*japonicus*	36.17'N, 137.48'E	JP	8	0.924	1.002	0.963	0.967	4.712	0.266	12	0.227	14	16.5	0.089	0.049	0.167	0.153	1	14	23	0.028
RVcoll17S377	*niger*	43.36'N, 28.08'E	BG	NA	0.875	0.947	0.911	0.912	5.045	0.231	9	0.228	5	15	0.075	0.040	0.156	0.095	5	17	13	0.027
RVcoll17S377	*niger*	43.36'N, 28.08'E	BG	8	0.889	0.924	0.906	0.895	3.703	0.231	5	0.222	3	7	0.058	0.043	0.167	0.087	3	12	16	0.029
RVcoll17S538	*niger*	43.82'N, 15.94'E	HR	NA	0.947	1.033	0.990	0.953	3.651	0.231	8	0.245	11	16	0.069	0.058	0.165	0.092	6	23	19	0.028
RVcoll17S538	*niger*	43.82'N, 15.94'E	HR	NA	0.860	0.924	0.892	0.886	3.844	0.219	9	0.225	10	11	0.078	0.049	0.144	0.110	5	14	18	0.025

CollectionCode	*Lasius* species	Lat/lon	Co-unt-ry	Ma-De	CW	CL	CS	SL	sq-PDCL	PoOc	n-Gen	EYE	n-GU	n-OCC	GU-HL	dCL-AN	MP6	Pn-HL	nSt	n-HT	n-SC	PLF
RVcoll17S383	*niger*	60.45'N, 22.29'E	FI	NA	1.039	1.077	1.058	0.964	4.103	0.287	11	0.247	11	19	0.095	0.064	0.167	0.110	6	23	14	0.030
RVcoll17S383	*niger*	60.45'N, 22.29'E	FI	NA	0.981	1.039	1.010	0.906	4.352	0.261	9	0.229	8	15	0.087	0.052	0.144	0.121	5	20	16	0.031
RVcoll17S383	*niger*	60.45'N, 22.29'E	FI	8	0.953	1.039	0.996	0.924	4.352	0.250	7	0.231	9	19	0.098	0.049	0.165	0.121	8	20	17	0.031
SS20B870	*niger*	44.13'N, 6.01'E	FR	8	0.981	1.010	0.996	0.950	4.121	0.211	9	0.242	5	13.5	0.072	0.055	0.173	0.130	5	19	18	0.028
SS20B870	*niger*	44.13'N, 6.01'E	FR	8	0.924	0.981	0.953	0.924	4.162	0.240	4	0.224	8	9	0.066	0.040	0.173	0.124	5	13	14	0.030
SS17B404	*niger*	43.86'N, 10.53'E	IT	NA	0.722	0.802	0.762	0.758	4.582	0.198	9	0.195	4	8.5	0.058	0.038	0.139	0.092	1	10	13	0.027
SS17B404	*niger*	43.86'N, 10.53'E	IT	8	0.771	0.860	0.815	0.808	3.916	0.193	8	0.211	6	15	0.066	0.038	0.144	0.115	3	12	10	0.027
SS17B404	*niger*	43.86'N, 10.53'E	IT	8	0.742	0.828	0.785	0.808	3.799	0.202	7	0.202	6	16	0.052	0.038	0.150	0.087	4	12	11	0.027
RVcoll17S201	*niger*	55.98'N, 37.41'E	RU	8	1.068	1.097	1.082	1.022	3.687	0.271	9	0.241	11	18	0.101	0.058	0.179	0.072	8	23	20	0.034
RVcoll17S201	*niger*	55.98'N, 37.41'E	RU	NA	1.039	1.068	1.054	1.005	4.247	0.261	6	0.244	10	17	0.072	0.046	0.179	0.110	6	5.5	12	0.036
RVcoll17S201	*niger*	55.98'N, 37.41'E	RU	8	0.924	0.981	0.953	0.929	3.575	0.241	7	0.229	10	19	0.069	0.049	0.153	0.107	0	14	12	0.031
RVcoll17S575	*niger*	47.39'N, 8.55'E	CH	9	0.906	0.979	0.942	0.947	3.708	0.240	8	0.215	8.5	13.5	0.087	0.052	0.162	0.118	4	16	18	0.028
RVcoll17S575	*niger*	47.39'N, 8.55'E	CH	9	0.973	1.039	1.006	0.964	3.657	0.273	8	0.221	4	13	0.075	0.046	0.167	0.127	4	12	20	0.032
SS19B672	*niger*	45.99'N, 8.92'E	CH	8	0.886	0.924	0.905	0.866	3.924	0.225	6	0.219	4	9.5	0.066	0.049	0.173	0.107	7	16	15	0.024
SS19B672	*niger*	45.99'N, 8.92'E	CH	8	0.895	0.924	0.909	0.880	3.698	0.219	7	0.215	7	15.5	0.058	0.046	0.173	0.115	4	19	21	0.027
SS19B672	*niger*	45.99'N, 8.92'E	CH	8	0.944	0.993	0.968	0.924	4.005	0.253	8	0.219	7	8	0.078	0.058	0.167	0.110	3	22	16	0.027
SS20B783	*niger*	51.46'N, 0.36'W	UK	8	0.981	1.010	0.996	0.967	4.005	0.225	6	0.237	11	17.5	0.069	0.052	0.165	0.098	4	18	17	0.025
SS20B783	*niger*	51.46'N, 0.36'W	UK	8	0.915	0.970	0.942	0.928	4.472	0.231	7	0.224	5	13	0.064	0.046	0.156	0.107	5	15	15	0.030
SS20B783	*niger*	51.46'N, 0.36'W	UK	8	0.895	0.981	0.938	0.924	3.708	0.211	7	0.227	5	19	0.052	0.049	0.159	0.107	5	16	14	0.028
SS20B783	*niger*	51.46'N, 0.36'W	UK	8	0.924	0.981	0.953	0.929	3.752	0.245	7	0.231	6	15	0.072	0.049	0.150	0.104	3	16	13	0.025
RSL16001	*niger*	NA	NA	8.5	0.722	0.779	0.751	0.788	3.799	0.192	8	0.179	6	14	0.061	0.035	0.141	0.101	3	18	15	0.036
RSL16001	*niger*	NA	NA	9	0.866	0.924	0.895	0.883	3.909	0.214	9	0.214	8	14	0.075	0.049	0.162	0.115	3	20	22	0.039
RSL16001	*niger*	NA	NA	8	0.745	0.808	0.776	0.751	3.915	0.195	8.5	0.189	7	14	0.064	0.038	0.144	0.107	3	15	18	0.041
RSL16001	*niger*	NA	NA	7.5	0.678	0.788	0.733	0.755	3.959	0.192	7.5	0.195	4	13	0.055	0.038	0.156	0.107	1	12	14.5	0.032
RSL16001	*niger*	NA	NA	8	0.701	0.765	0.733	0.759	3.942	0.196	7	0.180	6	11	0.052	0.038	0.144	0.092	3	14	16	0.034
RVcoll17S559	*platythorax*	43.6'N, 22.78'E	BG	NA	0.895	0.944	0.919	0.895	6.007	0.255	12	0.221	12	18	0.092	0.051	0.162	0.115	5	22	24	0.024
RVcoll17S559	*platythorax*	43.6'N, 22.78'E	BG	8	0.932	0.967	0.950	0.912	5.793	0.254	14.5	0.218	12	20	0.098	0.058	0.153	0.121	4	18	23	0.026

CollectionCode	*Lasius* species	Lat/lon	Co-unt-ry	Ma-De	CW	CL	CS	SL	sq-PDCL	PoOc	n-Gen	EYE	n-GU	n-OCC	GU-HL	dCL-AN	MP6	Pn-HL	nSt	n-HT	n-SC	PLF
RVcoll17S559	*platythorax*	43.6'N, 22.78'E	BG	8	0.878	0.924	0.901	0.866	4.308	0.242	15	0.198	10	18	0.115	0.040	0.165	0.118	4	20	21	0.021
RVcoll13T046	*platythorax*	55.83'N, 12.56'E	DK	9	1.088	1.085	1.087	1.031	5.493	0.286	18	0.242	20	21	0.101	0.055	0.182	0.153	5	39	25	0.026
RVcoll13T046	*platythorax*	55.83'N, 12.56'E	DK	8	1.068	1.097	1.082	0.987	5.762	0.286	11.5	0.237	13	19	0.092	0.049	0.147	0.165	5	34	36	0.034
RVcoll17S380	*platythorax*	60.48'N, 22.32'E	FI	NA	0.929	0.996	0.963	0.924	5.123	0.248	10	0.231	7	17	0.098	0.046	0.165	0.153	5	27	31	0.030
RVcoll17S380	*platythorax*	60.48'N, 22.32'E	FI	NA	1.010	1.074	1.042	0.981	6.110	0.289	13	0.238	8	18	0.104	0.055	0.173	0.170	1	22	23.5	0.030
RVcoll17S380	*platythorax*	60.48'N, 22.32'E	FI	8	0.981	1.010	0.996	0.976	4.541	0.270	10	0.231	9	18	0.087	0.052	0.176	0.170	6	21	23	0.029
RVcoll13T085	*platythorax*	47.4'N, 8.39'E	CH	8	1.039	1.068	1.054	0.987	5.373	0.260	15	0.227	25	25	0.101	0.049	0.173	0.173	5	28	26	0.028
RVcoll13T085	*platythorax*	47.4'N, 8.39'E	CH	8	0.981	1.010	0.996	0.996	5.123	0.257	12	0.212	14	19	0.092	0.049	0.182	0.147	3	30	28	0.027
RVcoll13T085	*platythorax*	47.4'N, 8.39'E	CH	8	0.976	1.010	0.993	0.987	4.379	0.241	12	0.214	9	19	0.104	0.049	0.173	0.165	2	28	29	0.026

*Paratypes

References

1. Schär, S. *et al.* Do Holarctic ant species exist? Trans-Beringian dispersal and homoplasy in the Formicidae. *J. Biogeogr.* **45**, 1917–1928 (2018).
2. Talavera, G., Espadaler, X. & Vila, R. Discovered just before extinction? The first endemic ant from the Balearic Islands (*Lasius balearicus* sp. nov.) is endangered by climate change. *J. Biogeogr.* **42**, 589–601 (2015).
3. Maruyama, M. *et al.* A DNA and morphology based phylogenetic framework of the ant genus *Lasius* with hypotheses for the evolution of social parasitism and fungiculture. *BMC Evol. Biol.* **8**, 237 (2008).
4. Steiner, F. M. *et al.* Phylogeny and bionomics of *Lasius austriacus* (Hymenoptera, Formicidae). *Insectes Sociaux* **51**, 24–29 (2004).
5. Viljakainen, L. & Pamilo, P. Selection on an antimicrobial peptide defensin in ants. *J. Mol. Evol.* **67**, 643–652 (2008).
6. Cremer, S. *et al.* The Evolution of Invasiveness in Garden Ants. *PLOS ONE* **3**, 1–9 (2008).
7. Laciny, A. Evidence of mermithism in a gyne of *Lasius niger* (Linnaeus, 1758)(Hymenoptera: Formicidae) from Burgenland, Austria. *Z. Arbeitsgemeinschaft Österr. Entomol.* **69**, 131–138 (2017).

8. Ratnasingham, S. & Hebert, P. D. N. BOLD: The Barcode of Life Data System (http://www.barcodinglife.org). *Mol. Ecol. Notes* **7**, 355–364 (2007).
9. Czechowski, W., Radchenko, A. & Czechowska, W. The ants (Hymenoptera: Formicidae) of Poland. (Museum and Institute of Zoology, Polish Academy of Sciences, 2002).
10. Seifert, B. A taxonomic revision of the Palaearctic members of the ant subgenus *Lasius s. str.* (Hymenoptera: Formicidae). *Abh. Berichte Naturkundemuseums Görlitz* **66**, 1–67 (1992).
11. Lebas, C., Galkowski, C., Blatrix, R. & Wegnez, P. *Fourmis d'Europe Occidentale. Le Premier Guide Complet d'Europe*. (Delachaux et Niestlé, 2016).
12. Douwes, P., Abenius, J., Cederberg, B. & Wahlstedt, U. *Nationalnyckeln til Sveriges Flora och Fauna. Steklar: Myror - getingar Hymenoptera: Formicidae - Vespidae*. (ArtDatabanken, Sveriges lantbruksuniversitet SLU, 2012).
13. Borowiec, L. & Salata, S. Ants of Greece–checklist, comments and new faunistic data (Hymenoptera: Formicidae). *Genus* **23**, 461–563 (2012).
14. Vonshak, M. & Ionescu-Hirsch, A. A checklist of the ants of Israel (Hymenoptera: Formicidae). *Isr. J. Entomol.* **39**, 33–55 (2009).
15. Bračko, G., Gomboc, M., Lupše, B., Marić, R. & Pristovšek, U. New faunistic data on ants (Hymenoptera: Formicidae) of the southern part of Montenegro. *Nat. Slov.* **16**, 41–51 (2014).
16. AntWeb. Available from http://www.antweb.org. (2021).
17. INaturalist. Available from https://www.inaturalist.org/. (2021).
18. Csösz, S., Marko, B. & Galle, L. Ants (Hymenoptera: Formicidae) of Stana Valley (Romania): Evaluation of the effectiveness of a myrmecological survey. *Entomol. Romanica* **6**, 121–126 (2001).
19. Khaustov, A. New species and records of the genus *Petalomium* (Acari: Heterostigmata: Pygmephoridae) from Crimea (Ukraine). *Acarina Русский Акарологический Журнал* **13**, 173–179 (2005).

20. Radchenko, A. G., Czechowska, W., Czechowski, W. & Siedlar, E. *Lasius niger* (L.) and *Lasius platythorax* Seifert (Hymenoptera, Formicidae)-a revolution in Polish myrmecological faunistics and zoocoenology? *Fragm. Faun.* **42**, 103–113 (1999).
21. Marko, B. Ants (Hymenoptera: Formicidae) of the “Sand Dunes of Foieni” protected area and its surroundings (Satu mare County, Romania), and a new species for the Romanian fauna. *Acta Sci. Transylvanica* **16**, 87–99 (2008).
22. Kiran, K. & Aktaç, N. The vertical distribution of the ant fauna (Hymenoptera: Formicidae) of the Samanli Mountains, Turkey. *Forest* **25**, 10 (1996).
23. Barech, G. *et al.* A first checklist and diversity of ants (Hymenoptera: Formicidae) of the saline dry lake Chott El Hodna in Algeria, a Ramsar Conservation Wetland. *Afr. Entomol.* **24**, 143–152 (2016).
24. Seifert, B. A taxonomic revision of the Palaearctic members of the subgenus *Lasius s. str.* (Hymenoptera, Formicidae). *Soil Org.* **92**, 15–86 (2020).
